# Ability of Radiomics in Differentiation of Anaplastic Oligodendroglioma From Atypical Low-Grade Oligodendroglioma Using Machine-Learning Approach

**DOI:** 10.3389/fonc.2019.01371

**Published:** 2019-12-17

**Authors:** Yang Zhang, Chaoyue Chen, Yangfan Cheng, Yuen Teng, Wen Guo, Hui Xu, Xuejin Ou, Jian Wang, Hui Li, Xuelei Ma, Jianguo Xu

**Affiliations:** ^1^Department of Neurosurgery, West China Hospital, Sichuan University, Chengdu, China; ^2^West China School of Medicine, West China Hospital, Sichuan University, Chengdu, China; ^3^Radiology Department, West China Hospital, Sichuan University, Chengdu, China; ^4^School of Computer Science, Nanjing University of Science and Technology, Nanjing, China; ^5^Department of Biotherapy, Cancer Center, West China Hospital, Sichuan University, Chengdu, China; ^6^State Key Laboratory of Biotherapy and Cancer Center, West China Hospital, Sichuan University and Collaborative Innovation Center for Biotherapy, Chengdu, China

**Keywords:** radiomics, machine learning, oligodendroglioma, anaplastic oligodendroglioma, magnetic resonance imaging, grading

## Abstract

**Objectives:** To investigate the ability of radiomics features from MRI in differentiating anaplastic oligodendroglioma (AO) from atypical low-grade oligodendroglioma using machine-learning algorithms.

**Methods:** A total number of 101 qualified patients (50 participants with AO and 51 with atypical low-grade oligodendroglioma) were enrolled in this retrospective, single-center study. Forty radiomics features of tumor images derived from six matrices were extracted from contrast-enhanced T1-weighted (T1C) images and fluid-attenuation inversion recovery (FLAIR) images. Three selection methods were performed to select the optimal features for classifiers, including distance correlation, least absolute shrinkage and selection operator (LASSO), and gradient boosting decision tree (GBDT). Then three machine-learning classifiers were adopted to generate discriminative models, including linear discriminant analysis, support vector machine, and random forest (RF). Receiver operating characteristic analysis was conducted to evaluate the discriminative performance of each model.

**Results:** Nine predictive models were established based on radiomics features from T1C images and FLAIR images. All of the classifiers represented feasible ability in differentiation, with AUC more than 0.840 when combined with suitable selection method. For models based on T1C images, the combination of LASSO and RF classifier represented the highest AUC of 0.904 in the validation group. For models based on FLAIR images, the combination of GBDT and RF classifier showed the highest AUC of 0.861 in the validation group.

**Conclusion:** Radiomics-based machine-learning approach could potentially serve as a feasible method in distinguishing AO from atypical low-grade oligodendroglioma.

## Introduction

Oligodendroglial tumors, one of the most common subtypes of gliomas, are classified into oligodendroglioma (grade II) and anaplastic oligodendroglioma (AO) (grade III) according to the 2016 World Health Organization (WHO) classification system (1). The clinical management and prognosis of oligodendrogliomas are closely relevant to the histopathological grade. AO is considered as the malignant tumor with aggressive behavior and requires radiotherapy and chemotherapy after the maximum safe resection, whereas patients with low-grade oligodendroglioma usually undergo less postoperative treatment and have better survival outcomes (2, 3). Therefore, the accurate preoperative assessment of tumor grade is clinically important for treatment planning and prognosis prediction. Magnetic resonance (MR) scan is recommended in pre-surgical evaluation of oligodendroglioma grade, as the contrast enhancement pattern is typically considered as the characteristics of high-grade glioma (2, 4). However, up to 50% of low-grade oligodendroglioma showed similar patterns with enhancement on MR imaging (MRI), making the discrimination from AO challenging in these cases (5).

Radiomics is an emerging field that can extract quantitative parameters from medical images to provide non-visual information calculated with mathematical formulas (6). Previous studies suggested that the combination of radiomics and machine-learning algorithms showed promising potential in differential diagnosis, pre-surgical grading, and prognosis prediction of intracranial tumors (7–10). However, it has never been applied in the grade prediction of oligodendrogliomas. Because radiomics could potentially reflect the underlying pathophysiology of lesions, we hypothesized that it might detect the differences that were difficult to obtain by visual inspection between AO and atypical oligodendroglioma (6, 11). Therefore, the purpose of the present study was to investigate the ability of radiomics-based machine learning technology in distinguishing AO from atypical low-grade oligodendroglioma. A set of radiomics parameters was extracted from MR images, and a series of discriminative models were established using different combinations of selection methods and machine-learning algorithms.

## Materials and Methods

### Patient Selection

**Figure 1:**
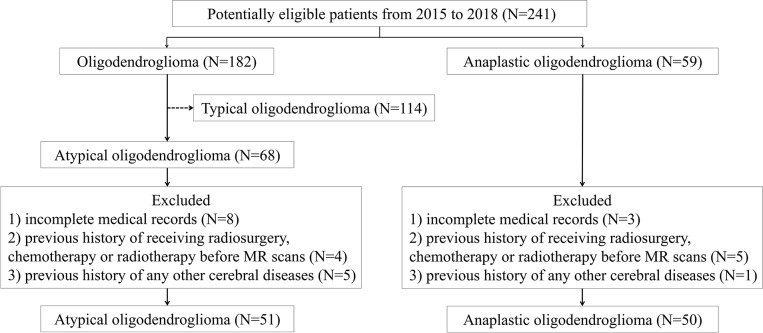
The flowchart of patient enrollment process. MR, magnetic resonance.

In this retrospective study, we screened our institutional database to review the patients who were diagnosed and treated at the neurosurgery department of our institution from January 2015 to December 2018. According to the 2016 WHO Classification of Tumors of the Central Nervous System, the presence of isocitrate dehydrogenase (IDH) mutation and 1p/19q codeletion is necessary for diagnosis of both oligodendroglioma and AO. Therefore, we carefully viewed the pathological reports and genetic testing results of all participants, ensuring that enrolled patients histopathologically and genetically met the 2016 WHO criteria. We initially selected 241 potentially eligible patients who were: (1) with pathological confirmation of oligodendroglioma (*N* = 182) or AO (*N* = 59); (2) with conclusive genetic testing results (presence of IDH mutation and 1p/19q codeletion); (3) with pre-therapeutic MR images. Among 182 patients with low-grade oligodendroglioma, 68 of them were selected as atypical oligodendroglioma defined as a low-grade oligodendroglioma with enhancement patterns on MRI. The exclusion criteria were as follows: (1) incomplete medical records (*N* = 11); (2) recorded history of receiving radiosurgery, chemotherapy, or radiotherapy before MR scans (*N* = 9); (3) previous history of any other cerebral diseases, such as stroke, subarachnoid hemorrhage (*N* = 6). The process of patient enrollment was shown in Figure 1. The clinical parameters, such as gender, age, Ki-67 labeling index of tumor, and days between MR scan and surgery were also recorded. This study was approved by the Ethics Committee of Sichuan University. The written informed consent was obtained from all participants enrolled in this study (written informed consent for patients under the age of 16 was obtained from parents or guardians).

### MRI Acquisition

**Figure 2:**
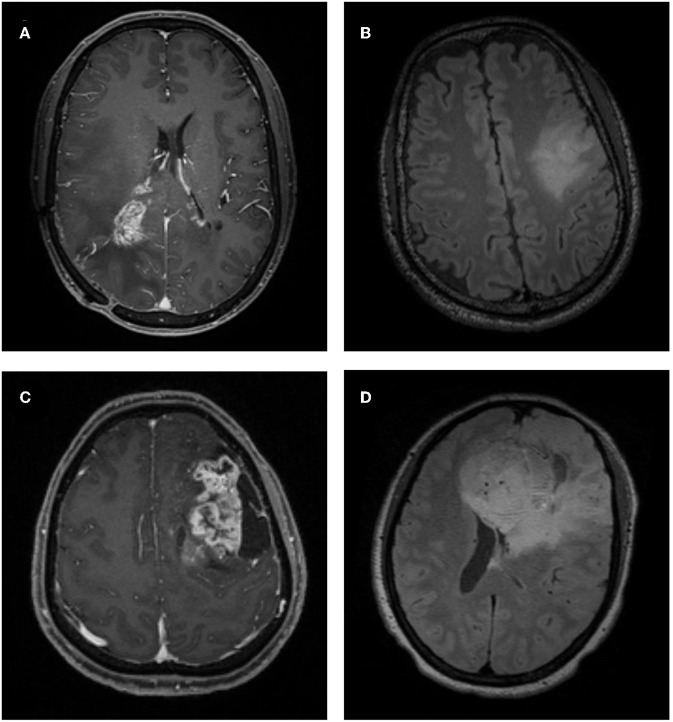
Examples of atypical low-grade oligodendroglioma and anaplastic oligodendroglioma on MRI. **(A)** A patient with atypical low-grade oligodendroglioma in contrast-enhanced T1-weighted (T1C) image. **(B)** A patient with atypical low-grade oligodendroglioma in fluid-attenuation inversion recovery (FLAIR) image. **(C)** A patient with anaplastic oligodendroglioma in T1C image. **(D)** A patient with anaplastic oligodendroglioma in FLAIR image.

All patients enrolled took MR scan *via* 3.0T GE SIGNA MRI scanner in our institution. In this study, contrast-enhanced T1-weighted (T1C) and fluid-attenuation inversion recovery (FLAIR) images were selected to perform texture analysis for the following reasons: first, they were the most important sequences in the diagnosis of oligodendrogliomas; second, the boundary of tumor and normal brain tissue should be clear and recognizable on images for precise delineation (Figure 2). The parameters of T1C image were as follows: TR/TE = 1,540/2.4 ms, slice thickness = 1 mm, axial FOV = 24 × 24 cm^2^, and data matrix = 256 × 256. The parameters of FLAIR image were as follows: TR/TE = 4,000/393 ms, slice thickness = 1 mm, axial FOV = 24 × 24 cm^2^, and data matrix = 516 × 516. Gadopentetate dimeglumine (0.1 mmol/kg) was used as the contrast agent for T1C sequence. MR images of all participants were collected with uniform standards through Picture Archiving and Communication Systems from our institutional radiology department.

### Texture Features Extraction

Texture features were extracted from MR images by two researchers together under the guidance of senior radiologists using LIFEx software (http://www.lifexsoft.org) (12). Following the instructions of the software, we manually contoured the regions of interest (ROI) on axial image slice by slice (obvious cystic area was not included in ROI considering the interference of cystic fluid). Disagreements between researchers on tumor boundary were addressed by consulting the senior radiologists. The edema band and adjacent structure invasion were carefully separated from the tumor tissue through the difference in contrast enhancement patterns in T1C images. Anatomic structures around the tumor were also recorded to help with delineation in FLAIR images. To ensure the accuracy of texture parameters, ROI was only drawn on the biggest one for tumors with clear boundary and on tumor-confirmed area for tumors with vague boundary. Even following this strategy, 12 FLAIR images were excluded because we were unable to delineate the tumor due to the interference of edema.

A total of 40 texture features were extracted from six matrices in the first or the second orders, including Histogram-based matrix, Shape-based matrix, Gray-level co-occurrence matrix (GLCM), Gray-level run length matrix (GLRLM), neighborhood gray-level dependence matrix (NGLDM), and Gray-level zone length matrix (GLZLM) (Supplementary Material 1). The association between features was assessed with Pearson's correlations (Supplementary Material 2).

### Model Establishment

**Figure 3:**
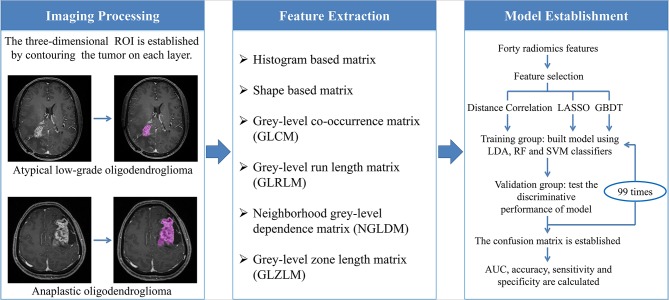
The workflow chart from image processing to model establishment. ROI, regions of interest; LASSO, least absolute shrinkage and selection operator; GBDT, gradient boosting decision tree; LDA, linear discriminant analysis; RF, random forest; SVM, support vector machine; AUC, area under the curve.

The optimal features needed to be selected first because the number of radiomics features was too large and not all of them were statistically significant. Features were chosen using three selection methods, namely, distance correlation, least absolute shrinkage and selection operator (LASSO), and gradient boosting decision tree (GBDT). Then three machine-learning classifiers were adopted to generate discriminative models, including random forest (RF), linear discriminant analysis (LDA), and support vector machine (SVM). LDA and SVM classifiers were chosen because they were representatives of linear and non-linear classification algorithms, respectively (13). Different from LDA or SVM, RF was considered the hybrid model of linear and non-linear classifiers by some researchers (14, 15). The patients were randomly divided into the training group and the validation group with the ratio of 4:1. The models were first trained with the training group and then applied to the independent validation group to test their discriminative performance, and this procedure was repeated for 100 cycles. A confusion matrix was established combining the histopathological results and predictions of models based on which the sensitivity, specificity, and accuracy were calculated. Area under the receiver operating characteristic curve (AUC) for both training group and validation group was also recorded to evaluate the discriminative ability of different models. The workflow from imaging processing to model establishment was shown in Figure 3.

## Results

### Patient Characteristics

A total number of 101 qualified patients (50 individuals with AO and 51 with atypical low-grade oligodendroglioma) were enrolled in the present study. The gender ratio of participants was 54:47 (male:female). The average ages of patients with AO and atypical low-grade oligodendroglioma were 47.1 and 38.7 years, respectively. The detailed characteristics of patients and lesions were summarized in Table 1.

**Table 1:** Characteristics of patients and lesions.

**Characteristics**	**Atypical low-grade oligodendroglioma (*n* = 51)**	**Anaplastic oligodendroglioma (*n* = 50)**
Age, *n* (%)		
0–20 years	5 (9.8)	1 (2.0)
21–40 years	22 (43.1)	15 (30.0)
41–60 years	19 (37.3)	23 (46.0)
61–80 years	5 (9.8)	11 (22.0)
Mean age (range) (year)	38.7 (7–71)	47.1 (16–76)
Gender, *n* (%)		
Male	29 (56.9)	25 (50.0)
Female	22 (43.1)	25 (50.0)
Ki-67 labeling index, *n* (%)		
<10%	35 (68.6)	9 (18.0)
≥10%	16 (31.4)	41 (82.0)
Average days between MR scan and surgery	9.4	7.9

*MR, magnetic resonance*.

### Model Assessment

A total of nine predictive models were built through the combination of three selection methods (distance correlation, LASSO, and GBDT) and three machine-learning classifiers (RF, LDA, and SVM). Radiomics features from T1C images and FLAIR images were introduced into models, respectively. All of the classifiers represented feasible discriminative ability with AUC more than 0.840 in the validation group when combined with the suitable selection method.

Among models using parameters from T1C images, the combination of LASSO and RF classifier (LASSO + RF) was proven to show the highest AUC of 0.904 in the validation group. Moreover, RF classifier seemed to be the optimal classification algorithm in differentiation for the reason that all RF-based models showed excellent performance with AUC over 0.920 in training group and 0.870 in the validation group (Table 2). For three models using the LDA classifier, receiver operating characteristic (ROC) analysis suggested that they all represented feasible discriminative ability, with AUC of 0.880 (distance correlation + LDA), 0.835 (LASSO + LDA), and 0.879 (GBDT + LDA) in the validation group (Table 3). For SVM-based models, only distance correlation + SVM showed feasible performance, with AUC of 0.866 in the validation group. Inadequate discriminative ability was observed in LASSO + SVM (AUC = 0.702 in the validation group) compared to other models, and overfitting was observed in GBDT + SVM (Table 4).

**Table 2:** Discriminative performance of models using RF classifier and different selection methods in distinguishing anaplastic oligodendroglioma from atypical low-grade oligodendroglioma in the training group and the validation group.

**Selection method**	**Training group**				**Validation group**			
	**AUC**	**Accuracy**	**Sensitivity**	**Specificity**	**AUC**	**Accuracy**	**Sensitivity**	**Specificity**
**T1C image**								
Distance correlation	0.927	0.928	0.959	0.901	0.874	0.876	0.925	0.825
LASSO	0.945	0.946	0.976	0.921	0.904	0.900	0.971	0.833
GBDT	0.959	0.960	0.984	0.939	0.896	0.895	0.952	0.838
**FLAIR image**								
Distance correlation	0.911	0.835	0.775	0.915	0.836	0.833	0.813	0.868
LASSO	0.946	0.863	0.844	0.882	0.855	0.756	0.780	0.725
GBDT	0.957	0.882	0.839	0.931	0.861	0.783	0.770	0.806

*RF, random forest; AUC, area under the curve; LASSO, least absolute shrinkage and selection operator; GBDT, gradient boosting decision tree; T1C, contrast-enhanced T1-weighted; FLAIR, fluid-attenuation inversion recovery*.

**Table 3:** Discriminative performance of models using LDA classifier and different selection methods in distinguishing anaplastic oligodendroglioma from atypical low-grade oligodendroglioma in the training group and the validation group.

**Selection method**	**Training group**				**Validation group**			
	**AUC**	**Accuracy**	**Sensitivity**	**Specificity**	**AUC**	**Accuracy**	**Sensitivity**	**Specificity**
**T1C image**								
Distance correlation	0.896	0.898	0.919	0.879	0.880	0.886	0.935	0.835
LASSO	0.928	0.929	0.949	0.911	0.835	0.829	0.926	0.748
GBDT	0.918	0.918	0.918	0.917	0.879	0.881	0.904	0.854
**FLAIR image**								
Distance correlation	0.866	0.796	0.727	0.900	0.843	0.783	0.740	0.887
LASSO	0.891	0.807	0.752	0.879	0.819	0.739	0.735	0.746
GBDT	0.943	0.862	0.836	0.889	0.848	0.817	0.802	0.841

*LDA, linear discriminant analysis; AUC, area under the curve; LASSO, least absolute shrinkage and selection operator; GBDT, gradient boosting decision tree; T1C, contrast-enhanced T1-weighted; FLAIR, fluid-attenuation inversion recovery*.

**Table 4:** Discriminative performance of models using SVM classifier and different selection methods in distinguishing anaplastic oligodendroglioma from atypical low-grade oligodendroglioma in the training group and the validation group.

**Selection method**	**Training group**				**Validation group**			
	**AUC**	**Accuracy**	**Sensitivity**	**Specificity**	**AUC**	**Accuracy**	**Sensitivity**	**Specificity**
**T1C**								
Distance correlation	0.885	0.889	0.981	0.829	0.866	0.857	0.989	0.760
LASSO	0.759	0.770	0.930	0.700	0.702	0.657	0.881	0.570
GBDT	1.000	1.000	1.000	1.000	/	/	/	/
**FLAIR**								
Distance correlation	0.904	0.738	0.650	0.965	0.860	0.772	0.715	0.953
LASSO	0.712	0.689	0.616	0.878	0.606	0.678	0.664	0.716
GBDT	1.000	1.000	1.000	1.000	/	/	/	/

*SVM, support vector machine; AUC, area under the curve; LASSO, least absolute shrinkage and selection operator; GBDT, gradient boosting decision tree; T1C, contrast-enhanced T1-weighted; FLAIR, fluid-attenuation inversion recovery; /, overfitting*.

Among models using parameters from FLAIR images, GBDT + RF was found to represent the highest AUC of 0.861 in the validation group. Besides, other two models using RF classifier also displayed feasible discriminative ability, with AUC of 0.836 (distance correlation + RF) and 0.855 (LASSO + RF) in the validation group (Table 2). For three models using LDA classifier, ROC analysis demonstrated that the AUC in the validation group were 0.843, 0.819, and 0.848, respectively (Table 3). Among SVM-based models, distance correlation + SVM represented the best performance in differentiation with AUC of 0.860 in the validation group. Overfitting was observed in GBDT + SVM again, indicating that this model might be unsuitable for the grade prediction (Table 4).

## Discussion

Accurate preoperative evaluation of tumor grade is important for treatment facilitation and prognosis prediction. Lacking specific blood biomarkers, MR scan is commonly performed to evaluate oligodendroglioma grade pre-surgically with high spatial resolution and tissue resolution. However, atypical low-grade oligodendroglioma with contrast enhancement could complicate the differentiation from AO (16). Searching for accurate diagnosis, the value of advanced MRI techniques in oligodendroglioma grading had been investigated in previous studies (17, 18). Nevertheless, these advanced imaging techniques require additional expense and platforms and are not routinely conducted for every patient in clinical work. In the current study, a series of radiomics parameters were extracted from conventional MR sequences and fed into machine-learning classifiers to differentiate AO from atypical low-grade oligodendroglioma. Several predictive models with suitable combination were proven to represent feasible ability in grade prediction. Given that both T1C and FLAIR sequences are routinely performed in clinical examination, machine learning-based radiomics could potentially serve as the imaging biomarkers to aid preoperative diagnosis.

Radiomics has been investigated in recent studies, implying that the parameters are associated with tumor histopathology and abnormal microenvironment. The texture features calculate the image characteristics from different aspects, statistically reflecting intratumoral heterogeneity, cellular density, and level of vascularization (19–21). This theory has been verified by previous researches that the shift of texture parameters was associated with irregularity in blood vessel distribution and intratumoral hypoxia (22, 23). Given that these biological procedures were regulated by DNA, texture parameters were also related to molecular pathologic characteristics of tumors, such as mutation status of IDH and Kirsten Ras (KRAS) (24, 25). As for oligodendrogliomas, AO is histologically characterized by high cellular density, nuclear atypia, and microvascular proliferation, which might contribute to its radiological characteristics, such as contrast enhancement. Thus, we hypothesized that texture parameters might help discriminate between grade II and III oligodendrogliomas.

Moreover, with analyzable statistics converted from images, the novel computer technology could be employed. Similar researches suggested that radiomics combined with machine-learning algorithms displayed promising potential in various fields, including differential diagnosis of glioblastoma, pre-surgical grading of glioma, and prediction of patient survival outcomes (8, 26–28). It is worth noting that previous studies primarily focused the value of radiomics in distinguishing low-grade glioma vs. high-grade glioma, whereas the possible different characteristics among the histological subtypes of glioma were not taken into consideration (29–31). However, the heterogeneity of different glioma subtypes might interfere with the accuracy of the models. Therefore, our study first applied radiomics in grade prediction of oligodendrogliomas, a specific, common subtype of gliomas. More importantly, we focused on the situation where visual inspection was not sufficient in discrimination, aiming to explore the ability of radiomics-based machine learning in differentiating AO from atypical low-grade oligodendroglioma.

Compared with previous studies on glioma grading, we employed more selection methods (distance correlation, LASSO, and GBDT) and machine learning classifiers (RF, LDA, and SVM), wishing to identify the optimal model with the best discriminative performance. The results indicated that all of the classifiers represented feasible discriminative ability when combined with suitable selection method, and RF-based models showed the best performance with highest AUC in the validation group. RF classifier is a robust classification algorithm that has represented high discriminative performance in many studies (24, 32, 33). The mechanism of RF classification algorithm is to build subtrees by using the training bootstrap samples and choose the classification with the most votes over all trees in the forest (34). On the other hand, the results also indicated that the selection method with different mechanisms may have effects on the performance of the models. Distance correlation is the representative of filter models that rank features based on certain characteristics and remove irrelevant features without classification algorithms, whereas LASSO and GBDT were representatives of embedded models that embed feature selection with classifier construction (35). However, we must note that most models represented similar diagnostic performance, and the differences in AUC may be partly attributed to the relatively small study cohort. Future studies with larger sample sizes are required to validate our results and further investigate the optimal model for grade prediction.

There were some limitations in the present study. First, this was a retrospective study; the selection bias was inevitable. Second, radiomics features were extracted from T1C and FLAIR sequences, whereas the value of features from other sequences like diffusion-weighted imaging (DWI) was unclear. Future studies are required to explore whether the features from other sequences could help improve the discriminative ability. Third, our models were not externally validated because this study was conducted in a single institution. However, the image processing and model establishment were conducted using open-source packages, providing the potential for researchers to verify our results in the future. Fourth, considering IDH and 1p/19q status could be reflected in texture parameters, it is reasonable to think that other molecular biomarkers may be associated with parameters. However, this point was not considered in the current study because of the relatively small sample size and single subtype of gliomas. Future larger studies are required to validate our results and to rectify the defects.

## Data Availability Statement

The raw data supporting the conclusions of this manuscript will be made available by the authors, without undue reservation, to any qualified researcher.

## Ethics Statement

The studies involving human participants were reviewed and approved by Ethics Committee of Sichuan University. Written informed consent to participate in this study was provided by the participants' legal guardian/next of kin. Written informed consent was obtained from the individual(s), and minor(s)' legal guardian/next of kin, for the publication of any potentially identifiable images or data included in this article.

## Author Contributions

YZ participated in study conception, image collection, feature extraction, and drafted the manuscript. CC participated in image collection, feature extraction, and manuscript revision. YC and YT participated in feature extraction and manuscript revision. WG extracted texture feature and performed statistical analysis. HX and XO collected image data and extracted texture feature. JW participated in model construction and statistical analysis. HL participated in data processing and statistical analysis. XM and JX participated in study conception and manuscript revision. All authors read and approved the submitted version.

### Conflict of Interest

The authors declare that the research was conducted in the absence of any commercial or financial relationships that could be construed as a potential conflict of interest.

## Supplementary Material

Supplementary Material 1 Original data of texture features extracted from T1C and FLAIR sequences.

Supplementary Material 2 Heat map showing Pearson's correlations between all pairs of features.
