# Supplementary material for: Ability of Radiomics in Differentiation of Anaplastic Oligodendroglioma From Atypical Low-Grade Oligodendroglioma Using Machine-Learning Approach
Source: Front Oncol. 2019 Dec 17;9:1371. doi: 10.3389/fonc.2019.01371 (PMC6929242; doi:10.3389/fonc.2019.01371)
Supplement: Supplementary Material 1 — Original data of texture features extracted from T1C and FLAIR sequences. [file Data_Sheet_1.pdf]

Supplementary Material 1

T1C

Patient	1	2	3	4	5	6	7	8
Grade (II=0,III=1)	0	0	0	0	0	0	0	0
minVaule	1246.7	27.594	70.167	87.544	569.2	364.16	347.65	63.409
meanValue	2737.6	259.01	319.89	309.27	1707.2	650.83	536.26	258.14
stdValue	324.26	61.25	50.309	45.806	247.12	64.962	53.989	36.226
maxValue	5744.2	828.6	703.54	610.17	3298.7	858.35	673.55	762.55
HISTO_Skewness	-0.089	0.5897	0.727	0.8354	-0.099	-0.861	-0.835	1.0925
HISTO_Kurtosis	5.5484	3.1162	5.4061	6.432	4.5798	4.0407	3.3528	13.928
HISTO_Entropy_log10	1.258	1.279	1.2982	1.3382	1.366	1.4926	1.5946	1.1039
HISTO_Energy	0.0698	0.0597	0.061	0.0571	0.053	0.0383	0.0319	0.0965
SHAPE_Volume	50.875	87.248	22.617	7.5612	88.486	22.163	16.386	3.4109
GLCM_Homogeneity	0.4422	0.5699	0.5085	0.3974	0.3364	0.3103	0.2918	0.5048
GLCM_Energy	0.0117	0.0115	0.01	0.0059	0.0043	0.0034	0.0023	0.0173
GLCM_Contrast	26.136	3.9358	10.14	17.645	31.226	45.298	77.252	5.7277
GLCM_Correlation	0.4169	0.9129	0.7855	0.7117	0.4798	0.5835	0.647	0.6916
GLCM_Entropy_log10	2.3112	2.1029	2.2248	2.4374	2.5536	2.629	2.9226	1.9477
GLCM_Dissimilarity	3.3035	1.3153	1.8821	2.908	4.0561	4.8378	6.0909	1.6688
GLRLM_SRE	0.8469	0.8052	0.8518	0.9136	0.9335	0.9469	0.9445	0.8706
GLRLM_LRE	5.1093	2.383	1.9103	1.4364	1.3401	1.2568	1.323	1.7744
GLRLM_LGRE	0.0028	0.0035	0.0018	0.0018	0.0022	0.0019	0.0021	0.0036
GLRLM_HGRE	494.48	391.17	700.17	801.79	775.67	1472.6	1509.2	351.55
GLRLM_SRLGE	0.0024	0.0028	0.0015	0.0017	0.0021	0.0018	0.0021	0.0032
GLRLM_SRHGE	419.36	319.01	604.8	736.54	725.84	1384.3	1417.8	309
GLRLM_LRLGE	0.0132	0.0084	0.0034	0.0025	0.0028	0.002	0.0027	0.0062
GLRLM_LRHGE	2500.6	892.12	1278.1	1129	1029.3	1901.9	2049.1	603.42
GLRLM_GLNU	7962.2	3938.8	1081.3	387.9	302.09	61.083	230.77	287.56
GLRLM_RLNU	94248	40568	12634	5576.2	4940.2	1427.7	6539.1	2255.9
GLRLM_RP	0.8013	0.7421	0.8028	0.885	0.9122	0.9295	0.9238	0.8262
NGLDM_Coarseness	5E-05	0.0003	0.0008	0.0015	0.0014	0.0056	0.0014	0.0031
NGLDM_Contrast	0.0242	0.0153	0.0166	0.0334	0.062	0.1608	0.2097	0.0291
NGLDM_Busyness	8.2149	3.029	0.5616	0.2854	0.3534	0.0708	0.2583	0.4616
GLZLM_SZE	0.3774	0.6636	0.6498	0.6324	0.6247	0.6743	0.6098	0.5985
GLZLM_LZE	27925	88034	10060	614.18	65.329	24.432	31.976	2411
GLZLM_LGZE	0.0033	0.005	0.0024	0.0032	0.0028	0.0032	0.0024	0.0058
GLZLM_HGZE	586.35	482.87	884.2	911.12	811.61	1311.7	1367.2	409.94
GLZLM_SZLGE	0.0011	0.0034	0.0018	0.0026	0.0016	0.0028	0.0015	0.0045
GLZLM_SZHGE	264.1	331.91	590.09	594.05	520.05	858.43	804.79	247.87
GLZLM_LZLGE	59.983	316.76	17.943	0.8679	0.1002	0.017	0.0322	8.2583
GLZLM_LZHGE	1E+07	3E+07	6E+06	450144	46961	45412	56775	724971
GLZLM_GLNU	308.29	186.27	78.934	58.731	78.625	22.348	74.888	27.402
GLZLM_ZLNU	1149.9	1778.4	854.62	584	717.46	304.56	1048.2	137.88
GLZLM_ZP	0.0513	0.0485	0.0951	0.2	0.3103	0.413	0.3742	0.1088

T1C

Patient	9	10	11	12	13	14	15	16
Grade (II=0,III=1)	0	0	0	0	0	0	0	0
minVaule	390.78	226.41	1914.4	151.72	640.15	39.021	1302.6	20.519
meanValue	1376	384.27	2787.9	314.81	1639.7	310.48	2072.7	192.86
stdValue	248.18	39.167	178.75	60.258	255.09	71.514	244.13	32.32
maxValue	2637.2	1065.6	3881.1	728.45	2744.9	1181.6	4188.8	542.35
HISTO_Skewness	-0.359	2.1189	0.2372	0.2045	-0.19	0.9891	1.9879	0.5731
HISTO_Kurtosis	3.5265	22.263	4.8535	3.4875	3.8508	8.1199	13.369	4.6128
HISTO_Entropy_log10	1.456	1.0411	1.3616	1.4159	1.4973	1.1962	1.2884	1.2009
HISTO_Energy	0.0421	0.1145	0.0518	0.044	0.0389	0.0775	0.0633	0.0749
SHAPE_Volume	43.844	94.429	18.901	16.23	56.934	124.21	83.47	61.968
GLCM_Homogeneity	0.3318	0.4885	0.3383	0.4255	0.3152	0.5443	0.3437	0.5453
GLCM_Energy	0.0034	0.0215	0.0043	0.0048	0.0028	0.0142	0.0054	0.0134
GLCM_Contrast	46.552	10.163	28.577	13.69	40.422	6.8717	44.468	4.9748
GLCM_Correlation	0.4875	0.3914	0.5529	0.8383	0.627	0.7701	0.2732	0.8303
GLCM_Entropy_log10	2.6985	1.9273	2.5071	2.4831	2.745	2.0748	2.4627	2.0618
GLCM_Dissimilarity	4.715	2.087	3.8194	2.5069	4.5907	1.5891	4.1522	1.4722
GLRLM_SRE	0.935	0.8461	0.9421	0.9006	0.9422	0.8242	0.9398	0.8263
GLRLM_LRE	1.3977	3.6754	1.279	1.5195	1.2902	2.1906	1.301	2.1323
GLRLM_LGRE	0.0019	0.0077	0.0018	0.0057	0.002	0.0054	0.0044	0.0024
GLRLM_HGRE	864.31	170.89	871.24	394.55	1016.5	268.45	341.39	488.02
GLRLM_SRLGE	0.0018	0.0066	0.0017	0.0052	0.0019	0.0045	0.0041	0.002
GLRLM_SRHGE	806.92	146.95	821.55	358.04	958.86	225.02	323	406.06
GLRLM_LRLGE	0.0025	0.0285	0.0021	0.0084	0.0024	0.0114	0.0056	0.0051
GLRLM_LRHGE	1216.2	576.9	1112.8	583.42	1303.6	557.26	432.81	1017.9
GLRLM_GLNU	389.21	3874.9	141.91	627.16	295.9	6553.4	445.29	3240.2
GLRLM_RLNU	8074.9	26414	2394.9	11195	6676.8	55908	6148.7	28385
GLRLM_RP	0.9115	0.8041	0.9235	0.8679	0.9233	0.7655	0.9201	0.771
NGLDM_Coarseness	0.0009	0.0002	0.003	0.001	0.0012	0.0001	0.0009	0.0003
NGLDM_Contrast	0.098	0.01	0.0741	0.0431	0.092	0.01	0.0438	0.01
NGLDM_Busyness	0.5087	5.351	0.1801	0.695	0.3204	5.2943	0.6557	1.9209
GLZLM_SZE	0.6251	0.4267	0.6538	0.635	0.6157	0.6472	0.6299	0.6383
GLZLM_LZE	54.503	24148	44.03	1646.7	30.985	81092	53.234	48860
GLZLM_LGZE	0.0026	0.0081	0.0024	0.008	0.0026	0.008	0.0048	0.0031
GLZLM_HGZE	835.42	263.29	903.22	482.12	1035.4	369.98	399.63	551.73
GLZLM_SZLGE	0.0019	0.0034	0.0019	0.0054	0.0018	0.0053	0.0032	0.0021
GLZLM_SZHGE	520.7	148.08	614.76	323.67	645.11	255.37	276.19	357.03
GLZLM_LZLGE	0.0697	171.12	0.0521	6.9255	0.0398	370.53	0.2105	113.07
GLZLM_LZHGE	49582	4E+06	42054	632880	30133	2E+07	15103	2E+07
GLZLM_GLNU	114.55	209.99	44.977	89.027	96.253	382.58	128.53	167.85
GLZLM_ZLNU	1198.4	615.02	413.16	964.18	1043.8	3529.6	968.54	1353.4
GLZLM_ZP	0.3213	0.0758	0.3484	0.1549	0.355	0.0794	0.339	0.0623

T1C

Patient	17	18	19	20	21	22	23	24
Grade (II=0,III=1)	0	0	0	0	0	0	0	0
minVaule	40.458	624	61.03	62.539	36.45	46	140.29	164.13
meanValue	251.34	2478.8	223.06	270.01	187.76	187.8	372.26	288.45
stdValue	45.744	399.7	46.163	52.246	44.519	35.6	61.773	52.019
maxValue	812	6257	797.52	637.96	508.47	450	1278.9	741.82
HISTO_Skewness	2.1061	0.92	0.8699	0.5591	-0.024	0.74	1.1382	2.1262
HISTO_Kurtosis	20.082	6.36	7.2006	4.3198	3.9448	5.92	12.298	14.471
HISTO_Entropy_log10	1.0936	1.25	1.2037	1.3667	1.3884	1.35	1.1324	1.3098
HISTO_Energy	0.1145	0.07	0.0753	0.0506	0.0496	0.06	0.0851	0.0609
SHAPE_Volume	33.326	127.32	117.87	14.275	55.975	37.072	28.925	6.9728
GLCM_Homogeneity	0.5331	0.379	0.5113	0.4278	0.4613	0.386	0.6833	0.44
GLCM_Energy	0.0235	0.006	0.0118	0.0056	0.007	0.006	0.0255	0.0072
GLCM_Contrast	13.36	22.328	6.7933	13.26	9.7019	25.006	2.2553	15.112
GLCM_Correlation	0.493	0.463	0.7828	0.7984	0.8452	0.572	0.8985	0.7855
GLCM_Entropy_log10	1.9701	2.39	2.1362	2.44	2.3719	2.46	1.778	2.3573
GLCM_Dissimilarity	2.0026	3.286	1.7355	2.5002	2.1949	3.468	0.8445	2.505
GLRLM_SRE	0.8334	0.919	0.844	0.8986	0.8799	0.914	0.7057	0.8901
GLRLM_LRE	2.511	1.43	1.9697	1.5298	1.8349	1.58	4.4117	1.6043
GLRLM_LGRE	0.0041	0.003	0.0062	0.0023	0.0047	0.003	0.0067	0.0094
GLRLM_HGRE	345.92	490.7	234.47	595.7	482.98	564.4	202.98	243.63
GLRLM_SRLGE	0.0036	0.002	0.0052	0.002	0.0042	0.002	0.0047	0.0083
GLRLM_SRHGE	293.03	454.1	201.62	539.43	427.67	518.9	147.98	221.58
GLRLM_LRLGE	0.0093	0.004	0.0125	0.0035	0.0085	0.004	0.0308	0.015
GLRLM_LRHGE	818.07	681.9	432.84	886.97	859.58	868.2	824.21	363
GLRLM_GLNU	1013.9	2179	6993.1	626.69	1316.9	1289.7	7087.8	528.4
GLRLM_RLNU	6507.5	26803	64038	9707.3	20575	19585	40701	6699.8
GLRLM_RP	0.7737	0.892	0.7931	0.8656	0.8382	0.884	0.6123	0.8545
NGLDM_Coarseness	0.0008	0	0.0001	0.001	0.0006	0	0.0002	0.0012
NGLDM_Contrast	0.0123	0.031	0.009	0.0343	0.0295	0.037	0.0032	0.0251
NGLDM_Busyness	0.7635	3	5.6945	0.5696	1.0898	1	3.8443	0.6098
GLZLM_SZE	0.6467	0.622	0.6249	0.6223	0.5269	0.539	0.5893	0.62
GLZLM_LZE	8086.4	998.3	86163	1292	3271.1	327.74	280572	1711.8
GLZLM_LGZE	0.0062	0.003	0.0069	0.0025	0.0052	0.003	0.0061	0.0099
GLZLM_HGZE	463.1	569.1	333.99	708.22	567.12	661.7	351.62	393.46
GLZLM_SZLGE	0.0042	0.002	0.0043	0.0017	0.0024	0.001	0.0031	0.006
GLZLM_SZHGE	329.6	369.1	219.52	454.18	321.92	386.7	239.16	274.17
GLZLM_LZLGE	26.789	2.4	483.18	3.009	8.9168	0.7	1730.2	11.612
GLZLM_LZHGE	2E+06	430175	2E+07	609846	1E+06	164830	5E+07	313086
GLZLM_GLNU	67.325	418.1	428.06	90.992	134.51	224.2	242.87	63.461
GLZLM_ZLNU	625.5	3037.3	3174.2	815.14	1024	1477.9	1453.8	550.25
GLZLM_ZP	0.128	0.23	0.0726	0.1557	0.1193	0.202	0.0326	0.1488

T1C

Patient	25	26	27	28	29	30	31	32
Grade (II=0,III=1)	0	0	0	0	0	0	0	0
minVaule	13.659	5	36.06	64	477.81	2132.2	31.803	56.89
meanValue	206.36	490.1	217.92	350.6	781.33	2750.1	127.62	214.7
stdValue	57.894	125.3	35.349	41.8	163.87	249.63	41.32	40.442
maxValue	583.17	1514	621.28	867	1216.4	4284	561.83	325.82
HISTO_Skewness	1.5096	1.64	1.4767	0.6	0.3366	1.3596	2.9617	-1.134
HISTO_Kurtosis	6.7604	9.17	10.901	9.17	2.0943	7.7142	13.891	5.4247
HISTO_Entropy_log10	1.3517	1.27	1.1464	1.11	1.7195	1.4305	1.0392	1.5294
HISTO_Energy	0.0549	0.07	0.0943	0.1	0.0206	0.0446	0.1421	0.04
SHAPE_Volume	75.125	72.476	36.115	52.33	7.99	7.7971	44.913	1.8484
GLCM_Homogeneity	0.5081	0.414	0.4182	0.504	0.2346	0.2744	0.5796	0.303
GLCM_Energy	0.0093	0.009	0.0135	0.017	0.0012	0.0032	0.0393	0.0031
GLCM_Contrast	8.3642	18.728	19.877	8.987	110.77	58.435	9.1955	43.012
GLCM_Correlation	0.8953	0.671	0.3342	0.564	0.7111	0.4765	0.8145	0.759
GLCM_Entropy_log10	2.2902	2.37	2.1878	2.01	3.0698	2.5984	1.8321	2.7099
GLCM_Dissimilarity	1.8503	2.843	2.9952	1.961	7.9414	5.5744	1.6124	4.7506
GLRLM_SRE	0.8467	0.898	0.8864	0.849	0.9608	0.963	0.7915	0.9496
GLRLM_LRE	1.9723	1.54	3.1145	2.42	1.2021	1.1708	2.9476	1.2264
GLRLM_LGRE	0.0025	0.003	0.0028	0.002	0.0051	0.0058	0.0085	0.0039
GLRLM_HGRE	556.14	480.6	436.68	558.3	931.03	416.14	192.17	1540.4
GLRLM_SRLGE	0.0021	0.003	0.0025	0.002	0.0048	0.0056	0.0064	0.0038
GLRLM_SRHGE	486.02	437.4	389.49	475.6	902.04	403.8	165.84	1462.6
GLRLM_LRLGE	0.0052	0.004	0.0085	0.005	0.0065	0.0066	0.0278	0.0043
GLRLM_LRHGE	979.55	705.1	1291.1	1331.9	1080	472.14	417.45	1890.2
GLRLM_GLNU	3146.1	6138.2	1397.6	3157.3	91.095	37.98	7952.3	78.659
GLRLM_RLNU	40886	66567	12453	25089	4052.1	783.27	40022	1775.7
GLRLM_RP	0.7938	0.864	0.8519	0.803	0.9469	0.9501	0.7143	0.9335
NGLDM_Coarseness	0.0003	0	0.0004	0	0.0023	0.0084	0.0001	0.0051
NGLDM_Contrast	0.023	0.025	0.0212	0.009	0.4678	0.1791	0.0135	0.1319
NGLDM_Busyness	1.9574	5	1.4199	2	0.3853	0.121	7.0523	0.0619
GLZLM_SZE	0.6532	0.65	0.4932	0.492	0.687	0.7083	0.5773	0.6655
GLZLM_LZE	31945	22194	1611.1	27739	12.852	8.3715	148162	22.895
GLZLM_LGZE	0.0023	0.003	0.0037	0.003	0.004	0.0068	0.0053	0.0062
GLZLM_HGZE	866.95	632.5	544.38	603.6	1044.4	476.75	438.51	1541.3
GLZLM_SZLGE	0.0017	0.002	0.0023	0.002	0.0027	0.0055	0.0026	0.0046
GLZLM_SZHGE	571.65	427.8	311.08	314.9	742.05	360.3	282.66	1032.9
GLZLM_LZLGE	93.86	57.9	3.8791	52.6	0.1522	0.0433	1490.1	0.0282
GLZLM_LZHGE	1E+07	9E+06	688477	1E+07	5995.8	2739.9	2E+07	35266
GLZLM_GLNU	226	687.4	105.17	171.2	49.022	18.067	278.97	23.199
GLZLM_ZLNU	2814.5	6728.9	531.76	709.8	1024.2	214.88	2244.5	363.46
GLZLM_ZP	0.0932	0.171	0.1253	0.073	0.4993	0.511	0.0768	0.4078

T1C

Patient	33	34	35	36	37	38	39	40
Grade (II=0,III=1)	0	0	0	0	0	0	0	0
minVaule	97.811	167.7	108.01	999.02	284.64	5.88	811.85	136.02
meanValue	222.68	308.31	205.87	2263.8	446.22	290.7	2382.8	283.01
stdValue	39.371	25.296	23.306	312.13	43.997	41.557	811.72	33.68
maxValue	814	429.05	558.57	5257.3	607.77	599.94	7567.3	428.54
HISTO_Skewness	2.5728	0.0199	1.2864	-0.102	-0.492	1.1832	1.2368	-0.35
HISTO_Kurtosis	16.95	5.8972	12.2	5.8231	2.6544	9.6335	6.1407	5.8884
HISTO_Entropy_log10	1.063	1.3711	1.1091	1.2722	1.5237	1.1893	1.4448	1.4322
HISTO_Energy	0.1126	0.0549	0.0949	0.0655	0.034	0.0866	0.0423	0.0483
SHAPE_Volume	65.636	16.128	67.168	38.044	70.056	17.696	175.81	0.6092
GLCM_Homogeneity	0.6146	0.3936	0.5808	0.4046	0.3591	0.4877	0.3656	0.2827
GLCM_Energy	0.0295	0.0059	0.0207	0.0072	0.0033	0.0185	0.0039	0.0048
GLCM_Contrast	4.4935	26.493	4.4807	16.349	35.824	13.533	39.559	50.034
GLCM_Correlation	0.8045	0.6684	0.7924	0.6161	0.766	0.6039	0.6801	0.3724
GLCM_Entropy_log10	1.8167	2.4904	1.9132	2.3519	2.7356	2.0886	2.6611	2.4317
GLCM_Dissimilarity	1.2425	3.3732	1.3288	2.9054	4.2173	2.3941	4.0814	5.2046
GLRLM_SRE	0.7832	0.918	0.7939	0.9206	0.9117	0.85	0.9201	0.9634
GLRLM_LRE	3.5627	1.5292	2.9238	1.4778	1.6869	5.0424	1.5129	1.1656
GLRLM_LGRE	0.0087	0.0013	0.0058	0.0043	0.0016	0.0013	0.0097	0.0035
GLRLM_HGRE	160.85	1262.4	225.38	402.06	1125.3	1019.2	306.57	1123.7
GLRLM_SRLGE	0.0066	0.0012	0.0047	0.004	0.0015	0.0011	0.0088	0.0034
GLRLM_SRHGE	133.11	1160.7	183.1	369.69	1021.4	874.48	288.25	1084.5
GLRLM_LRLGE	0.0345	0.0018	0.0171	0.0059	0.0026	0.0059	0.0163	0.0036
GLRLM_LRHGE	461.16	1910.7	607.9	595.69	1932.5	4697.5	410.01	1300.4
GLRLM_GLNU	5405	382.05	3586.7	294.57	1170.2	3401.1	512.98	28.653
GLRLM_RLNU	31381	5910.6	24226	3819.4	29147	32721	10219	554.69
GLRLM_RP	0.7067	0.8902	0.7261	0.8943	0.8825	0.7902	0.8904	0.9513
NGLDM_Coarseness	0.0002	0.0013	0.0003	0.0018	0.0004	0.0002	0.0008	0.0129
NGLDM_Contrast	0.0062	0.0435	0.006	0.0632	0.0987	0.0161	0.0801	0.1252
NGLDM_Busyness	4.5403	0.2388	2.7039	0.6056	1.158	1.9084	1.0918	0.034
GLZLM_SZE	0.5233	0.5789	0.5441	0.5727	0.473	0.495	0.6169	0.7387
GLZLM_LZE	101041	155.2	82993	102.14	209.77	21988	274.13	7.686
GLZLM_LGZE	0.0066	0.0018	0.009	0.0055	0.0019	0.0016	0.0086	0.0053
GLZLM_HGZE	325.12	1319.1	327.17	395.48	1038.1	1273.8	428.84	1165.5
GLZLM_SZLGE	0.0031	0.001	0.006	0.0028	0.0009	0.0007	0.0055	0.005
GLZLM_SZHGE	200.77	784.42	200.62	231.96	476.23	648.96	293.5	883.16
GLZLM_LZLGE	945.82	0.1404	458.28	0.2778	0.2328	24.146	5.5158	0.012
GLZLM_LZHGE	1E+07	181580	2E+07	43673	272415	2E+07	28612	8216.8
GLZLM_GLNU	192.85	74.298	131.25	67.799	231.25	161.09	125.17	12.465
GLZLM_ZLNU	1017.2	592.62	725.21	406.01	1602.3	956.15	1287.1	173.92
GLZLM_ZP	0.0539	0.2364	0.0476	0.2567	0.1868	0.0772	0.262	0.5375

T1C

Patient	41	42	43	44	45	46	47	48
Grade (II=0,III=1)	0	0	0	0	0	0	0	0
minVaule	97.39	52.694	122	39.709	126.56	55.714	44.244	921.95
meanValue	320.42	287.96	232.2	370.28	286.74	248.66	220.48	1566.5
stdValue	54.971	47.001	39	62.914	40.273	32.717	27.72	443.04
maxValue	994.65	812.76	649	889.11	523.31	357.3	383.36	4472.5
HISTO_Skewness	1.6305	1.454	2.99	0.6236	0.8904	-0.318	-0.309	1.8786
HISTO_Kurtosis	10.735	11.879	19.42	4.2867	5.3584	3.9515	4.9323	8.0029
HISTO_Entropy_log10	1.1565	1.1736	1.15	1.2487	1.4023	1.4494	1.3225	1.393
HISTO_Energy	0.0849	0.0826	0.1	0.0691	0.0492	0.0417	0.0584	0.0525
SHAPE_Volume	44.603	37.576	22.969	24.197	49.37	68.715	38.445	54.685
GLCM_Homogeneity	0.5743	0.5493	0.462	0.636	0.3434	0.3495	0.4261	0.3541
GLCM_Energy	0.0185	0.0157	0.018	0.019	0.0041	0.0036	0.0071	0.0064
GLCM_Contrast	5.9718	7.6623	26.848	3.3975	30.038	32.55	14.697	51.139
GLCM_Correlation	0.7944	0.7407	0.315	0.9162	0.6433	0.6176	0.6899	0.5261
GLCM_Entropy_log10	1.9743	2.0317	2.09	1.9647	2.599	2.65	2.3714	2.4882
GLCM_Dissimilarity	1.4273	1.5838	2.956	1.076	3.9081	3.9941	2.6586	4.5892
GLRLM_SRE	0.8021	0.8188	0.874	0.743	0.9355	0.9258	0.9005	0.9256
GLRLM_LRE	2.4499	2.2015	2.23	3.7976	1.3298	1.5712	1.656	1.4536
GLRLM_LGRE	0.0043	0.0028	0.007	0.0018	0.0019	0.0008	0.0011	0.0211
GLRLM_HGRE	296.45	436.37	225.9	685.43	741.72	1763.7	1167.3	223.63
GLRLM_SRLGE	0.0034	0.0023	0.006	0.0014	0.0018	0.0007	0.001	0.0193
GLRLM_SRHGE	245.58	363.05	203.3	518.54	697.43	1631.9	1051.4	215.7
GLRLM_LRLGE	0.011	0.0061	0.016	0.0068	0.0024	0.0011	0.0017	0.0326
GLRLM_LRHGE	656.69	916.85	433.6	2438.6	963.74	2781.2	1934.5	268.44
GLRLM_GLNU	3184	2565.9	1270.3	4826.4	436.74	599.28	886.18	204.15
GLRLM_RLNU	24026	20359	10729	39965	7722.3	12323	12350	3443.6
GLRLM_RP	0.7356	0.7613	0.827	0.6559	0.9143	0.8997	0.8652	0.8986
NGLDM_Coarseness	0.0003	0.0003	0	0.0002	0.001	0.0007	0.0007	0.0017
NGLDM_Contrast	0.0082	0.0082	0.023	0.0079	0.0588	0.0648	0.0256	0.122
NGLDM_Busyness	2.2111	1.4033	2	1.818	0.4789	0.4187	0.4849	0.7395
GLZLM_SZE	0.6358	0.6893	0.576	0.5972	0.6201	0.5689	0.559	0.6808
GLZLM_LZE	51233	39343	5556.9	190955	56.922	89.338	1261.7	225.85
GLZLM_LGZE	0.0043	0.0035	0.007	0.005	0.0022	0.0011	0.0016	0.0162
GLZLM_HGZE	461.62	628.49	393.4	726.57	819.59	1754.4	1181.7	384.05
GLZLM_SZLGE	0.0029	0.0022	0.003	0.004	0.0015	0.0008	0.0011	0.0099
GLZLM_SZHGE	307.98	461.51	265.4	416.96	527.19	983.31	660.22	302.02
GLZLM_LZLGE	237.26	104.21	33.9	346.52	0.1044	0.0558	1.1068	6.0804
GLZLM_LZHGE	1E+07	2E+07	934417	1E+08	34804	157079	1E+06	12068
GLZLM_GLNU	181.66	126.24	97	128.42	117.01	144.96	129.59	53.004
GLZLM_ZLNU	1455.7	1347.2	690.2	1291.5	1125.8	1266.3	954.75	633.47
GLZLM_ZP	0.0715	0.072	0.133	0.0341	0.3185	0.2588	0.1782	0.3231

T1C

Patient	49	50	51	52	53	54	55	56
Grade (II=0,III=1)	0	0	0	1	1	1	1	1
minVaule	45.005	60.254	0	165.47	206.5	1354.4	1159.3	348.09
meanValue	216	388.87	250.26	283.42	448.14	1794.1	2042.9	801.75
stdValue	69.849	87.686	80.266	57.044	84.113	172.36	283.77	179.28
maxValue	851.47	1774.3	981.44	523.54	822.9	2234.2	3381.3	1334
HISTO_Skewness	3.1862	4.48	1.1454	0.3832	0.314	-0.008	0.4975	0.0966
HISTO_Kurtosis	15.84	40.759	6.363	2.9348	3.4268	2.5222	4.3849	3.208
HISTO_Entropy_log10	1.0939	0.9706	1.2942	1.6004	1.5481	1.6849	1.503	1.6428
HISTO_Energy	0.1271	0.1441	0.062	0.028	0.0339	0.0235	0.0386	0.0282
SHAPE_Volume	297.14	69.792	42.308	4.3317	3.4153	3.9138	12.197	8.9338
GLCM_Homogeneity	0.537	0.6295	0.4325	0.2142	0.2879	0.1963	0.2497	0.2408
GLCM_Energy	0.0304	0.04	0.0078	0.002	0.002	0.0022	0.003	0.0017
GLCM_Contrast	24.78	7.448	16.77	121.72	43.197	139.86	82.642	160.03
GLCM_Correlation	0.6203	0.6559	0.6857	0.4266	0.6942	0.5531	0.3166	0.4509
GLCM_Entropy_log10	2.0086	1.7117	2.3607	2.8532	2.8414	2.7204	2.6437	2.9903
GLCM_Dissimilarity	2.5442	1.3156	2.763	8.7142	4.9789	9.165	6.5573	9.1829
GLRLM_SRE	0.816	0.75	0.8877	0.9658	0.957	0.9791	0.9705	0.9586
GLRLM_LRE	3.6046	3.344	1.8292	1.2229	1.1917	1.092	1.1353	1.2294
GLRLM_LGRE	0.0063	0.0068	0.0064	0.0073	0.0047	0.0046	0.0038	0.0082
GLRLM_HGRE	253.7	186.8	323.51	576.76	734.66	1215.2	744.47	1039.5
GLRLM_SRLGE	0.0051	0.005	0.0057	0.007	0.0045	0.0045	0.0037	0.0076
GLRLM_SRHGE	220.77	148.78	295.04	560.19	705.93	1192.2	725.45	999.91
GLRLM_LRLGE	0.0254	0.0241	0.0123	0.0099	0.0059	0.0049	0.004	0.0131
GLRLM_LRHGE	668.06	531.64	519.61	670.64	861.94	1316.6	829.79	1244.9
GLRLM_GLNU	5772.1	5649.2	1363.7	137.28	148.09	15.32	39.42	94.781
GLRLM_RLNU	34414	22812	17300	4567.2	3937.7	620.18	962.06	3083.8
GLRLM_RP	0.7387	0.6609	0.8433	0.9506	0.943	0.972	0.9608	0.9433
NGLDM_Coarseness	0.0001	0.0002	0.0004	0.0019	0.0025	0.0118	0.007	0.0026
NGLDM_Contrast	0.0257	0.0049	0.0292	0.2129	0.1241	0.4939	0.207	0.337
NGLDM_Busyness	6.3673	4.2124	1.6356	0.39	0.2338	0.0487	0.0938	0.2066
GLZLM_SZE	0.5894	0.6527	0.5662	0.6774	0.6745	0.7883	0.7334	0.6729
GLZLM_LZE	55466	129364	2243.6	9.4056	13.046	3.171	5.2904	10.901
GLZLM_LGZE	0.005	0.0061	0.0057	0.0057	0.0041	0.0052	0.0048	0.0038
GLZLM_HGZE	475.14	385.03	464.92	649.04	782.32	1262.4	806.46	1118.8
GLZLM_SZLGE	0.0027	0.0042	0.0026	0.0035	0.0026	0.0044	0.0043	0.0015
GLZLM_SZHGE	305.62	279.07	284.58	459.55	539.38	1018.3	620.36	774.37
GLZLM_LZLGE	364.47	921.7	14.576	0.1138	0.0556	0.011	0.0136	0.2905
GLZLM_LZHGE	9E+06	2E+07	386220	3502.6	8228.1	3567.7	3368.9	9109.8
GLZLM_GLNU	313.99	223.64	199.29	74.651	65.915	10.381	21.292	44.432
GLZLM_ZLNU	2461.4	1611.3	1317.2	1121.9	922.73	266.7	317.05	726.36
GLZLM_ZP	0.1065	0.0622	0.163	0.5102	0.4688	0.6865	0.5909	0.4809

T1C

Patient	57	58	59	60	61	62	63	64
Grade (II=0,III=1)	1	1	1	1	1	1	1	1
minVaule	154.19	57.004	193.11	289.07	1424.3	543.19	1034	228.96
meanValue	511.87	190.15	314.53	490.55	1770.9	813.8	2123.4	647.93
stdValue	95.17	63.284	47.406	89.5	170.17	136.24	356.32	170.89
maxValue	734.72	393.63	453.73	780.65	2119.4	1492.4	3679.3	1587
HISTO_Skewness	-0.943	0.146	-0.151	0.4333	-0.117	1.0424	0.1353	1.4946
HISTO_Kurtosis	3.604	2.302	2.7477	2.8974	2.0749	4.724	2.9414	6.8803
HISTO_Entropy_log10	1.5861	1.6621	1.662	1.6488	1.7327	1.5237	1.535	1.4484
HISTO_Energy	0.0317	0.0239	0.0252	0.0258	0.0209	0.0362	0.0334	0.0459
SHAPE_Volume	4.425	1.294	2.327	0.5651	0.2997	11.211	3.2972	10.555
GLCM_Homogeneity	0.3106	0.2088	0.1939	0.2294	0.1162	0.2967	0.2414	0.2489
GLCM_Energy	0.0022	0.0013	0.0018	0.0024	0.0151	0.003	0.0032	0.0031
GLCM_Contrast	37.711	110.5	134.66	82.456	898.14	89.945	116.23	92.748
GLCM_Correlation	0.829	0.6169	0.4751	0.727	-0.209	0.4544	0.1182	0.2446
GLCM_Entropy_log10	2.8457	2.9575	2.8271	2.6693	1.949	2.762	2.6956	2.6815
GLCM_Dissimilarity	4.511	8.113	9.09	6.9891	25.705	6.4243	8.0872	7.0532
GLRLM_SRE	0.948	0.9767	0.9808	0.9772	0.9838	0.9464	0.9577	0.9638
GLRLM_LRE	1.2376	1.0998	1.0902	1.1017	1.0887	1.396	1.7095	1.1745
GLRLM_LGRE	0.0014	0.0063	0.0045	0.0053	0.0158	0.0078	0.0047	0.0047
GLRLM_HGRE	1690	817.33	1055.4	851.23	1298.1	447.31	801.92	481.77
GLRLM_SRLGE	0.0014	0.0061	0.0044	0.0052	0.0155	0.0073	0.0046	0.0046
GLRLM_SRHGE	1591.7	802.28	1035.5	832.2	1278.9	429.66	769.65	469.3
GLRLM_LRLGE	0.0017	0.0072	0.0048	0.0056	0.0171	0.0113	0.0101	0.0054
GLRLM_LRHGE	2143.3	879.84	1145.8	931.82	1402.7	568.06	1299.4	539.2
GLRLM_GLNU	132.78	31.926	27.458	14.942	5.6658	161.26	114.68	61.502
GLRLM_RLNU	3713.3	1259.3	1039.4	547.14	262.79	4042.4	3264.2	1251
GLRLM_RP	0.9311	0.9689	0.9738	0.9692	0.9768	0.9239	0.943	0.9512
NGLDM_Coarseness	0.0033	0.0069	0.0074	0.0178	0.0371	0.0016	0.0018	0.0043
NGLDM_Contrast	0.1309	0.4338	0.3903	0.3068	0.6944	0.1767	0.2421	0.1891
NGLDM_Busyness	0.0954	0.1226	0.0738	0.0423	0.0154	0.5149	0.3432	0.1767
GLZLM_SZE	0.6552	0.7723	0.7938	0.7523	0.8172	0.6269	0.5524	0.7277
GLZLM_LZE	20.316	3.6761	2.8458	3.3106	2.5833	28.442	15.764	8.738
GLZLM_LGZE	0.0019	0.0052	0.005	0.006	0.0152	0.007	0.0032	0.0053
GLZLM_HGZE	1529.2	875.53	1063.3	877.46	1350.4	597.53	869.41	573.95
GLZLM_SZLGE	0.0015	0.0031	0.0043	0.0051	0.0106	0.0039	0.0014	0.0043
GLZLM_SZHGE	968.79	697.32	848.77	682.08	1140.9	427.82	517	455.58
GLZLM_LZLGE	0.0162	0.0298	0.0113	0.0143	0.0354	0.2762	0.094	0.0374
GLZLM_LZHGE	41467	2360.9	2922.6	2709.1	3181.4	7267.3	10997	2869.6
GLZLM_GLNU	51.697	21.743	19.36	9.7374	4.0098	53.525	43.741	27.402
GLZLM_ZLNU	735.01	507.33	463.46	207.85	127.09	639.19	418.5	377.73
GLZLM_ZP	0.4053	0.664	0.7069	0.6611	0.7312	0.3578	0.3848	0.5358

T1C

Patient	65	66	67	68	69	70	71	72
Grade (II=0,III=1)	1	1	1	1	1	1	1	1
minVaule	1200.3	192.56	177.59	98.189	987.45	172.72	3082.6	341.15
meanValue	2453.7	244.3	360.98	136.54	2009	636.1	4511.5	549.74
stdValue	433.58	21.695	64.241	14.827	586.05	141.57	544.16	89.518
maxValue	4317.2	320.13	561.34	192.87	3334.3	1007.8	6229.5	899.2
HISTO_Skewness	0.0913	0.6798	0.5224	0.5954	0.3448	-0.347	0.2965	0.5047
HISTO_Kurtosis	3.7605	2.9486	2.5754	3.2654	1.7916	3.0593	2.8312	3.2618
HISTO_Entropy_log10	1.5466	1.6125	1.6108	1.5973	1.7124	1.6346	1.6422	1.6127
HISTO_Energy	0.034	0.0281	0.0285	0.0296	0.0217	0.0269	0.0266	0.0289
SHAPE_Volume	6.3729	4.5485	5.0807	3.4314	5.4815	1.4738	3.3484	15.793
GLCM_Homogeneity	0.2557	0.2314	0.4233	0.3456	0.2072	0.2327	0.2121	0.2692
GLCM_Energy	0.0019	0.0017	0.0033	0.0032	0.002	0.0015	0.0019	0.0017
GLCM_Contrast	75.644	92.23	12.729	21.856	196	89.923	177.73	81.46
GLCM_Correlation	0.5636	0.5989	0.9404	0.8284	0.4788	0.6106	0.2332	0.5271
GLCM_Entropy_log10	2.8429	2.8951	2.6922	2.6297	2.8048	2.9298	2.7996	2.9488
GLCM_Dissimilarity	6.3818	7.3435	2.5777	3.5299	10.618	7.1344	9.8886	6.4091
GLRLM_SRE	0.9642	0.9688	0.9051	0.9544	0.9687	0.9717	0.9748	0.9575
GLRLM_LRE	1.1578	1.1466	1.5737	1.2329	1.1657	1.1204	1.1253	1.2152
GLRLM_LGRE	0.0042	0.0029	0.0019	0.0029	0.0052	0.004	0.003	0.0051
GLRLM_HGRE	770.25	824.81	1101.5	803.91	1069.3	1413.1	992.55	711.12
GLRLM_SRLGE	0.0041	0.0029	0.0017	0.0028	0.005	0.0039	0.0029	0.0049
GLRLM_SRHGE	744.87	803.47	1009.6	771.39	1041.7	1372.4	965.41	687.09
GLRLM_LRLGE	0.0047	0.0034	0.0029	0.0034	0.0061	0.0043	0.0033	0.0065
GLRLM_LRHGE	882.74	923.48	1639	969.77	1207.8	1587.2	1133.2	828.56
GLRLM_GLNU	62.735	64.762	436.82	74.965	27.396	41.082	64.245	220.22
GLRLM_RLNU	1699.1	2143.4	12400	2294.9	1190.2	1428.5	2277.2	6892.1
GLRLM_RP	0.9529	0.9582	0.87	0.9391	0.9574	0.9626	0.9658	0.9421
NGLDM_Coarseness	0.0049	0.0035	0.0016	0.0066	0.0072	0.0063	0.0048	0.0012
NGLDM_Contrast	0.1623	0.3011	0.0763	0.1069	0.7359	0.2554	0.2413	0.2326
NGLDM_Busyness	0.1068	0.2173	0.3527	0.0967	0.1276	0.0657	0.1267	0.5628
GLZLM_SZE	0.6896	0.7039	0.5304	0.6695	0.7008	0.754	0.7389	0.6804
GLZLM_LZE	6.6946	5.6129	656.41	11.391	5.506	4.4692	3.9879	12.292
GLZLM_LGZE	0.0045	0.0031	0.0019	0.0033	0.0051	0.0045	0.0032	0.0047
GLZLM_HGZE	817.03	899.43	1320.2	882.28	1176.9	1392.7	951.4	815.63
GLZLM_SZLGE	0.003	0.0023	0.0009	0.0024	0.0037	0.0033	0.0025	0.0029
GLZLM_SZHGE	587.29	683.31	711.84	619.16	866.63	1034.7	690.92	585.11
GLZLM_LZLGE	0.0216	0.0163	1.2866	0.0267	0.0318	0.0115	0.0098	0.0742
GLZLM_LZHGE	4660.1	3748.3	410623	7800.6	4618	6415.6	4477.1	6035.8
GLZLM_GLNU	32.673	35.34	80.61	33.183	14.581	24.883	41.991	102.98
GLZLM_ZLNU	462.72	628.06	894.15	526.97	340.55	520.87	797.12	1663.2
GLZLM_ZP	0.5379	0.5695	0.1838	0.4659	0.5615	0.6189	0.6301	0.4771

T1C

Patient	73	74	75	76	77	78	79	80
Grade (II=0,III=1)	1	1	1	1	1	1	1	1
minVaule	197.06	3223.8	16.156	175.12	179.17	198.27	102.54	192.09
meanValue	324.22	4588.3	270.58	326.28	307.33	312.91	245.63	250.73
stdValue	51.649	845.45	144.71	52.452	33.846	69.583	59.037	23.739
maxValue	469.81	6515.1	708.93	528.51	415.25	525.74	574.15	338.96
HISTO_Skewness	0.1477	0.4204	0.666	-0.317	-0.282	0.1337	1.3075	0.2784
HISTO_Kurtosis	2.6275	2.3818	2.7893	3.1631	3.8243	2.1272	6.9211	3.1508
HISTO_Entropy_log10	1.6774	1.6925	1.6885	1.5614	1.5626	1.6646	1.4677	1.6124
HISTO_Energy	0.0242	0.0235	0.0236	0.0325	0.0341	0.0249	0.0423	0.0288
SHAPE_Volume	3.2459	1.4561	0.8865	0.597	0.8732	2.0019	4.0593	1.332
GLCM_Homogeneity	0.2163	0.1214	0.2098	0.239	0.3638	0.2702	0.2617	0.256
GLCM_Energy	0.002	0.0068	0.0018	0.003	0.0034	0.0028	0.003	0.0023
GLCM_Contrast	314.97	622.12	110.85	77.814	23.231	104.49	101.63	80.498
GLCM_Correlation	0.0467	-0.047	0.6227	0.4955	0.8417	0.4953	0.2625	0.5991
GLCM_Entropy_log10	3.0007	2.2057	2.8244	2.604	2.6799	2.7797	2.6929	2.7688
GLCM_Dissimilarity	13.287	19.547	8.0937	6.7085	3.5247	7.4914	7.1359	6.7496
GLRLM_SRE	0.9371	0.9867	0.9777	0.9726	0.9339	0.9504	0.9559	0.9675
GLRLM_LRE	2.3131	1.058	1.0943	1.1175	1.3928	1.6726	1.2416	1.2161
GLRLM_LGRE	0.003	0.0195	0.0122	0.0052	0.0017	0.0184	0.0058	0.0081
GLRLM_HGRE	1072.2	1006.3	761.92	867.76	1328.2	730.75	465.42	789.04
GLRLM_SRLGE	0.0029	0.0188	0.0117	0.0051	0.0017	0.0169	0.0056	0.0076
GLRLM_SRHGE	1006.1	995.39	750.2	843.38	1242.7	700.75	448.03	764.97
GLRLM_LRLGE	0.0075	0.0232	0.0142	0.0054	0.0022	0.0465	0.0069	0.0125
GLRLM_LRHGE	2419.5	1050.1	812.48	972.97	1845.8	1024	557.28	946.69
GLRLM_GLNU	247.36	4.6609	18.973	16.854	160.09	89.195	77.035	48.243
GLRLM_RLNU	9562.6	194.11	764.03	488.41	4073.8	3328.5	1672.4	1561.9
GLRLM_RP	0.9213	0.9827	0.9703	0.9638	0.9083	0.9258	0.94	0.9523
NGLDM_Coarseness	0.0006	0.0186	0.0122	0.0178	0.003	0.0043	0.0032	0.0058
NGLDM_Contrast	0.5645	1.8952	0.479	0.2872	0.0891	0.2796	0.1886	0.225
NGLDM_Busyness	1.0356	0.046	0.0896	0.0383	0.1241	0.1804	0.2332	0.1134
GLZLM_SZE	0.3742	0.8795	0.7713	0.7661	0.5857	0.6241	0.667	0.6957
GLZLM_LZE	45.164	2.097	3.5108	4.0578	51.469	34.282	12.86	8.2721
GLZLM_LGZE	0.0027	0.0117	0.0098	0.0069	0.0023	0.0052	0.0065	0.0049
GLZLM_HGZE	1118.4	1045.5	854.93	841.71	1376.5	927.54	542.89	814.52
GLZLM_SZLGE	0.0009	0.0051	0.0062	0.0064	0.0013	0.0014	0.0041	0.0025
GLZLM_SZHGE	431.27	942.19	709.15	630.28	823.11	633.1	400.83	578.47
GLZLM_LZLGE	0.1389	0.0819	0.0525	0.0117	0.0517	1.7169	0.0566	0.1105
GLZLM_LZHGE	43687	1797.2	2096.6	3666.6	66843	10741	4880.2	6136.1
GLZLM_GLNU	66.464	3.4848	12.047	10.179	43.139	35.828	30.279	24.84
GLZLM_ZLNU	539.02	120.92	305.63	188.28	519.56	516.83	361.88	414.91
GLZLM_ZP	0.2567	0.8088	0.6635	0.636	0.3066	0.3636	0.4466	0.5243

T1C

Patient	81	82	83	84	85	86	87	88
Grade (II=0,III=1)	1	1	1	1	1	1	1	1
minVaule	330.59	2344	160.12	272.5	231.63	513.64	175.92	135
meanValue	724.83	3014.5	452.17	338.97	536.7	1204.7	826.31	190.44
stdValue	129.63	291.73	101.77	39.998	111.78	227.22	194.67	32.292
maxValue	1163.3	5302.1	754.01	546.6	1055.8	1827.6	1232	339.72
HISTO_Skewness	-0.028	2.1302	0.1833	1.8941	0.2007	-0.735	-0.535	1.5866
HISTO_Kurtosis	2.6862	13.143	2.9273	7.9411	3.2584	3.2924	2.258	6.3269
HISTO_Entropy_log10	1.6007	1.3354	1.622	1.4564	1.5429	1.6248	1.6178	1.5256
HISTO_Energy	0.0284	0.0565	0.0281	0.0443	0.0333	0.0286	0.0283	0.0378
SHAPE_Volume	1.8096	27.524	0.3823	0.3921	3.0146	10.699	2.2055	0.8705
GLCM_Homogeneity	0.272	0.3012	0.2065	0.3035	0.2799	0.254	0.3003	0.2453
GLCM_Energy	0.0017	0.0044	0.0029	0.0043	0.002	0.0016	0.0022	0.0025
GLCM_Contrast	66.578	52.617	105.7	47.82	44.488	109.24	61.056	120.94
GLCM_Correlation	0.6719	0.2792	0.4725	0.7058	0.6693	0.5292	0.7618	0.4455
GLCM_Entropy_log10	2.8933	2.4886	2.5939	2.4848	2.8213	3.0064	2.8641	2.7213
GLCM_Dissimilarity	5.8802	5.0155	8.0422	4.8812	5.0907	7.4966	5.3759	7.5693
GLRLM_SRE	0.9589	0.9569	0.9791	0.9538	0.9591	0.9601	0.9545	0.9644
GLRLM_LRE	1.1824	1.1988	1.0876	1.2099	1.1822	1.2186	1.211	1.1545
GLRLM_LGRE	0.0036	0.0098	0.004	0.0151	0.004	0.0035	0.0021	0.0116
GLRLM_HGRE	1053.1	268.09	1146.8	351.77	659.68	1290.8	1716.1	427.06
GLRLM_SRLGE	0.0034	0.0094	0.0039	0.0147	0.0038	0.0033	0.002	0.0112
GLRLM_SRHGE	1013.5	258.86	1125.6	341.3	631.82	1239.5	1626.8	416.83
GLRLM_LRLGE	0.0042	0.0115	0.0041	0.0169	0.0046	0.0047	0.0023	0.013
GLRLM_LRHGE	1226.7	310.05	1235	398	782.7	1566.5	2134.7	470.34
GLRLM_GLNU	54.421	82.93	12.39	21.526	100.31	288.68	75.166	31.934
GLRLM_RLNU	1728.1	1335.4	420.73	441.1	2726.9	9243.2	2416	779.6
GLRLM_RP	0.9455	0.9432	0.9724	0.9385	0.9456	0.9446	0.9388	0.9532
NGLDM_Coarseness	0.0059	0.004	0.0178	0.019	0.0035	0.0008	0.0046	0.0082
NGLDM_Contrast	0.1995	0.1303	0.3921	0.2082	0.1514	0.2979	0.2498	0.2785
NGLDM_Busyness	0.0928	0.2884	0.035	0.0747	0.1953	0.5084	0.0846	0.1237
GLZLM_SZE	0.6972	0.6797	0.8056	0.6897	0.682	0.6581	0.709	0.6992
GLZLM_LZE	10.65	9.9907	3.0031	11.683	12.667	10.388	20.177	6.7773
GLZLM_LGZE	0.0036	0.0105	0.0049	0.022	0.0045	0.0028	0.0026	0.0135
GLZLM_HGZE	1102.8	315.07	1180.3	440.16	651.34	1293.3	1539.5	523.07
GLZLM_SZLGE	0.0022	0.0062	0.0045	0.0197	0.0033	0.0016	0.0018	0.0112
GLZLM_SZHGE	779.04	239.18	976.01	324.11	444.17	844.6	1056.1	410
GLZLM_LZLGE	0.0285	0.08	0.0079	0.1165	0.032	0.0482	0.0157	0.0657
GLZLM_LZHGE	9176.6	2136.6	3195.5	2449.5	9092.8	13096	43436	1958.6
GLZLM_GLNU	27.864	34.664	8.2586	8.627	48.041	137.37	32.26	15.495
GLZLM_ZLNU	453.96	320.79	194.68	112.12	669.22	2049.4	634.72	219.49
GLZLM_ZP	0.4966	0.4756	0.707	0.4755	0.4846	0.474	0.472	0.5407

T1C

Patient	89	90	91	92	93	94	95	96
Grade (II=0,III=1)	1	1	1	1	1	1	1	1
minVaule	2914.1	386.83	2502.9	1480.8	203.6	398.2	2286.3	905.43
meanValue	3717.1	572.93	5832.6	1830.4	435	443.24	3127.5	1782.5
stdValue	280.22	45.179	1067.9	197.38	115.57	21.712	542.64	579.87
maxValue	4562.2	778.77	7773.1	2873.7	649.04	529.45	5310	3923.3
HISTO_Skewness	0.3508	0.035	-0.398	2.6459	-0.322	0.928	1.4642	1.1568
HISTO_Kurtosis	3.4887	4.7694	2.3919	11.672	1.9377	3.8205	5.0919	3.5761
HISTO_Entropy_log10	1.5663	1.4606	1.6648	1.36	1.7543	1.5858	1.5506	1.5755
HISTO_Energy	0.0339	0.0422	0.0244	0.06	0.0189	0.0312	0.0364	0.0351
SHAPE_Volume	1.2706	2.0657	7.8089	10.239	1.6906	2.985	8.4701	26.366
GLCM_Homogeneity	0.1627	0.3114	0.2471	0.2922	0.1646	0.2073	0.2583	0.273
GLCM_Energy	0.0064	0.0037	0.0019	0.0068	0.0031	0.0023	0.0037	0.0029
GLCM_Contrast	192.27	50.208	84.316	81.458	215.66	141.96	127.21	97.981
GLCM_Correlation	0.1503	0.4979	0.7666	0.1447	0.1915	0.2658	0.527	0.6484
GLCM_Entropy_log10	2.2367	2.5981	2.8235	2.3261	2.5694	2.7206	2.5374	2.8147
GLCM_Dissimilarity	11.107	5.1738	6.8105	5.7032	11.62	8.9879	7.5435	6.8756
GLRLM_SRE	0.9885	0.9444	0.9674	0.9565	0.9832	0.9767	0.9659	0.9547
GLRLM_LRE	1.046	1.334	1.1493	1.2232	1.097	1.1116	1.1596	1.232
GLRLM_LGRE	0.0066	0.0024	0.0044	0.0075	0.0121	0.007	0.0145	0.0084
GLRLM_HGRE	1122.5	1009.8	1831.3	366.16	1414	622.74	475.47	530.99
GLRLM_SRLGE	0.0066	0.0023	0.0041	0.0072	0.0118	0.0067	0.0141	0.0079
GLRLM_SRHGE	1110.3	953.47	1762.1	356.75	1390	612.2	465.73	516.8
GLRLM_LRLGE	0.0067	0.0029	0.0053	0.009	0.0144	0.0083	0.0163	0.0106
GLRLM_LRHGE	1171	1339.5	2151.4	413.36	1544.7	670.96	519.96	598.03
GLRLM_GLNU	6.6326	100.79	25.05	39.825	16.618	30.282	19.024	115.58
GLRLM_RLNU	192.16	2137.3	951.57	614.76	844.08	924.94	488.96	3063.4
GLRLM_RP	0.9851	0.924	0.9566	0.9416	0.9757	0.9683	0.9546	0.9392
NGLDM_Coarseness	0.028	0.0033	0.0103	0.0082	0.0134	0.0065	0.0152	0.0025
NGLDM_Contrast	0.7074	0.1015	0.3677	0.2613	0.642	0.3571	0.3914	0.3197
NGLDM_Busyness	0.0254	0.1319	0.0395	0.1276	0.041	0.1254	0.0834	0.4441
GLZLM_SZE	0.8755	0.6194	0.6855	0.6755	0.8141	0.7744	0.7171	0.6889
GLZLM_LZE	1.753	25.935	5.5814	11.867	2.7805	3.5863	6.7508	20.045
GLZLM_LGZE	0.0077	0.0028	0.0034	0.0087	0.0085	0.0057	0.0159	0.0074
GLZLM_HGZE	1135.8	1034.3	1691.7	502.69	1428.8	694.9	598.95	695.97
GLZLM_SZLGE	0.0075	0.0014	0.0012	0.0068	0.0041	0.0034	0.0112	0.0046
GLZLM_SZHGE	1003.6	657.62	1120.2	405.96	1185.8	574.23	490	528.6
GLZLM_LZLGE	0.0085	0.0362	0.0217	0.082	0.0447	0.0296	0.0728	0.2056
GLZLM_LZHGE	1924.1	25713	12064	2540.3	3887.9	1716.2	1931.7	3997.5
GLZLM_GLNU	4.8193	34.528	14.326	14.006	12.614	18.943	8.5987	41.964
GLZLM_ZLNU	120.33	345.83	263.11	142.72	399.96	374	146.99	759.74
GLZLM_ZP	0.8259	0.3702	0.5574	0.4662	0.7197	0.664	0.5538	0.4746

T1C

Patient	97	98	99	100	101
Grade (II=0,III=1)	1	1	1	1	1
minVaule	315.79	97.257	2456.6	415.38	669.28
meanValue	599.74	287.55	4483.6	503.71	931.47
stdValue	181.04	52.073	719.73	26	130
maxValue	1166.8	522.69	6922.6	592.55	1355.4
HISTO_Skewness	0.5099	-0.579	0.2665	-0.296	0.8333
HISTO_Kurtosis	2.5267	3.5711	3.2909	2.8643	3.6371
HISTO_Entropy_log10	1.6923	1.4882	1.5943	1.5829	1.651
HISTO_Energy	0.0224	0.0392	0.031	0.0302	0.027
SHAPE_Volume	9.6548	4.3723	9.3158	12.698	3.3244
GLCM_Homogeneity	0.2447	0.3286	0.218	0.3231	0.201
GLCM_Energy	0.0018	0.003	0.0023	0.002	0.002
GLCM_Contrast	176.76	27.872	115.71	25.024	145.25
GLCM_Correlation	0.5217	0.7691	0.3939	0.8503	0.5393
GLCM_Entropy_log10	3.038	2.6894	2.7212	2.8392	2.7746
GLCM_Dissimilarity	9.7949	3.9804	8.0852	3.8698	9.201
GLRLM_SRE	0.9503	0.945	0.9748	0.9418	0.981
GLRLM_LRE	1.4853	1.2924	1.1233	1.2672	1.0894
GLRLM_LGRE	0.0166	0.0025	0.0033	0.0018	0.0084
GLRLM_HGRE	685.35	908.62	983.66	1140.5	771.55
GLRLM_SRLGE	0.0153	0.0024	0.0033	0.0018	0.0082
GLRLM_SRHGE	659.84	857.71	961.29	1075.4	757.77
GLRLM_LRLGE	0.0378	0.003	0.0036	0.0022	0.0099
GLRLM_LRHGE	862.67	1179.5	1091.5	1439	834.29
GLRLM_GLNU	208.7	152.57	27.035	355.96	29.915
GLRLM_RLNU	8567.1	3426	826.56	10197	1057.7
GLRLM_RP	0.9244	0.925	0.9654	0.923	0.974
NGLDM_Coarseness	0.001	0.0033	0.0081	0.001	0.0074
NGLDM_Contrast	0.4385	0.0944	0.3341	0.1284	0.3877
NGLDM_Busyness	1.0499	0.1631	0.0702	0.4438	0.1043
GLZLM_SZE	0.6125	0.5964	0.7451	0.6652	0.7885
GLZLM_LZE	35.944	26.494	4.3902	66.482	2.8436
GLZLM_LGZE	0.0058	0.0031	0.0038	0.0026	0.0065
GLZLM_HGZE	889.31	898.1	1026.5	1155.7	791.77
GLZLM_SZLGE	0.002	0.0018	0.0033	0.002	0.0043
GLZLM_SZHGE	595.26	532.84	782.85	773.63	640.56
GLZLM_LZLGE	2.1644	0.0417	0.0118	0.0791	0.0327
GLZLM_LZHGE	8018.4	24763	3960	74555	2120.3
GLZLM_GLNU	82.162	50.394	15.768	113.17	21.298
GLZLM_ZLNU	1321.7	489.25	292.41	1724.7	461.18
GLZLM_ZP	0.3709	0.3481	0.6253	0.3278	0.7021

FLAIR							
Patient	1	2	3	4	5	6	7
Grade (II=0,III=1)	0	0	0	0	0	0	0
minValue	2986.216	209.6854	251.6907	369.7826	150.7122	218.0972	2344.425
meanValue	4541.669	324.8345	443.6393	499.476	235.4428	363.3386	2695.856
stdValue	281.2554	27.8586	47.44111	24.31062	15.1083	44.52901	117.4892
maxValue	5161.817	407.7741	581.6628	550.3487	288.7237	477.5089	3109.241
HISTO_Skewness	-1.06177	-0.12436	0.024897	-0.83865	-0.52507	-0.59356	0.046226
HISTO_Kurtosis	5.19935	2.748383	3.140283	5.54042	4.486891	3.117159	3.429041
HISTO_Entropy_log10	1.492157	1.557431	1.572163	1.496894	1.444375	1.622059	1.562915
HISTO_Energy	0.038013	0.031266	0.031561	0.035985	0.044276	0.028964	0.031639
SHAPE_Volume	7.144935	11.83208	5.950538	2.7096	0.739821	3.325296	1.544888
GLCM_Homogeneity	0.268127	0.30938	0.257713	0.232779	0.378746	0.218695	0.185341
GLCM_Energy	0.002682	0.00216	0.002331	0.00493	0.004415	0.002087	0.005559
GLCM_Contrast	89.32174	43.19459	109.3819	91.56938	16.71217	166.1418	139.6327
GLCM_Correlation	0.262444	0.722038	0.356772	0.205676	0.802819	0.406806	0.319838
GLCM_Entropy_log10	2.717559	2.810143	2.839808	2.379193	2.528045	2.772988	2.312503
GLCM_Dissimilarity	6.696101	4.71756	7.806422	7.187128	3.002992	9.396355	9.470585
GLRLM_SRE	0.963671	0.95439	0.953668	0.979124	0.932814	0.971464	0.983581
GLRLM_LRE	1.198497	1.237369	1.351765	1.101183	1.329989	1.140557	1.068752
GLRLM_LGRE	0.001555	0.001186	0.001319	0.004301	0.001119	0.0038	0.007551
GLRLM_HGRE	2200.105	1500.3	1503.586	2233.941	1629.177	1439.769	992.1013
GLRLM_SRLGE	0.001454	0.001149	0.001268	0.004164	0.001074	0.003527	0.007346
GLRLM_SRHGE	2115.199	1431.176	1431.459	2187.69	1517.357	1397.905	977.0929
GLRLM_LRLGE	0.001986	0.001376	0.001659	0.004856	0.001339	0.00491	0.008379
GLRLM_LRHGE	2665.851	1866.739	2054.045	2457.848	2179.495	1652.89	1053.782
GLRLM_GLNU	145.9881	123.8598	211.8291	18.20401	157.6779	45.24543	11.25293
GLRLM_RLNU	3565.619	3565.621	6151.943	482.5202	3049.161	1471.049	341.187
GLRLM_RP	0.949849	0.938071	0.933947	0.971319	0.910099	0.961986	0.978809
NGLDM_Coarseness	0.002123	0.002837	0.001105	0.014363	0.00397	0.006259	0.017789
NGLDM_Contrast	0.140632	0.137461	0.183125	0.382997	0.051455	0.269702	0.429568
NGLDM_Busyness	0.136396	0.1503	0.332859	0.042735	0.087574	0.063504	0.041835
GLZLM_SZE	0.674445	0.647117	0.605899	0.787283	0.589912	0.711424	0.805556
GLZLM_LZE	8.273123	14.67901	18.03393	3.033058	65.50758	4.434116	2.186813
GLZLM_LGZE	0.0012	0.001604	0.001499	0.003296	0.002001	0.00283	0.00594
GLZLM_HGZE	2104.216	1468.362	1460.496	2249.386	1579.695	1412.892	1013.11
GLZLM_SZLGE	0.000543	0.001301	0.00081	0.001127	0.00168	0.001485	0.002853
GLZLM_SZHGE	1382.059	931.1156	867.6637	1775.641	910.8157	996.2152	826.867
GLZLM_LZLGE	0.011891	0.012661	0.016229	0.012569	0.043153	0.021702	0.019008
GLZLM_LZHGE	19467.36	21465.77	28446.42	6667.62	107444.8	6674.424	2070.385
GLZLM_GLNU	71.17046	53.80945	82.25274	12.42149	41.39899	26.37079	8.802198
GLZLM_ZLNU	871.277	725.1932	972.2315	209.1708	386.0219	460.2053	164.7656
GLZLM_ZP	0.506127	0.438044	0.391178	0.694073	0.298268	0.59951	0.752066

FLAIR							
Patient	8	9	10	11	12	13	14
Grade (II=0,III=1)	0	0	0	0	0	0	0
minValue	475.0338	4261.662	427.0234	4619.272	441.2159	339.2877	4105.167
meanValue	617.9307	5108.943	503.6792	5808.636	562.8424	445.0585	4827.865
stdValue	48.75498	277.8687	35.15664	322.8098	29.64903	29.76586	237.2191
maxValue	729.8167	5774.786	599.1851	6847.986	639.44	501.3472	5276.654
HISTO_Skewness	-0.29786	-0.12409	0.553181	0.196208	-0.11396	-0.73142	-0.55407
HISTO_Kurtosis	2.683814	2.941111	2.483559	3.346536	2.758921	3.016806	3.125751
HISTO_Entropy_log10	1.683644	1.648048	1.685596	1.562889	1.583429	1.638806	1.621682
HISTO_Energy	0.023651	0.026848	0.023456	0.032176	0.029369	0.027021	0.027598
SHAPE_Volume	6.880412	3.894979	3.049839	9.947867	2.02596	1.767701	2.236186
GLCM_Homogeneity	0.222635	0.221371	0.219908	0.238151	0.302214	0.221124	0.153025
GLCM_Energy	0.001584	0.003055	0.001994	0.002298	0.002168	0.003329	0.0081
GLCM_Contrast	180.1819	94.40702	266.2484	73.17739	59.69089	107.8428	311.7661
GLCM_Correlation	0.387298	0.549563	0.334264	0.444034	0.663833	0.619679	-0.1373
GLCM_Entropy_log10	2.978766	2.580582	2.768014	2.73946	2.787625	2.55271	2.143432
GLCM_Dissimilarity	9.845408	7.662029	11.22464	6.668535	5.335797	7.917926	13.99501
GLRLM_SRE	0.965344	0.976862	0.974354	0.970437	0.956725	0.979877	0.984244
GLRLM_LRE	1.290559	1.102595	1.130529	1.140135	1.217681	1.101745	1.066512
GLRLM_LGRE	0.002988	0.003108	0.003781	0.002054	0.001272	0.002141	0.007702
GLRLM_HGRE	1464.255	1457.441	1016.373	1287.416	1672.799	1922.736	1765.815
GLRLM_SRLGE	0.002902	0.00308	0.003671	0.002025	0.001237	0.002125	0.007691
GLRLM_SRHGE	1409.98	1423.369	993.2573	1250.557	1600.331	1882.172	1738.849
GLRLM_LRLGE	0.003705	0.003229	0.004304	0.00219	0.001441	0.002218	0.007749
GLRLM_LRHGE	1953.48	1612.441	1129.256	1464.115	2038.856	2128.009	1880.744
GLRLM_GLNU	71.35773	15.98292	39.24582	44.66074	79.34337	24.23904	5.558956
GLRLM_RLNU	2821.578	565.4333	1579.41	1300.136	2448.378	857.5553	195.7226
GLRLM_RP	0.949909	0.969505	0.964701	0.960165	0.941815	0.972171	0.97929
NGLDM_Coarseness	0.002705	0.015589	0.006307	0.006695	0.004448	0.012681	0.023931
NGLDM_Contrast	0.410522	0.359115	0.377028	0.187653	0.152049	0.279125	1.088503
NGLDM_Busyness	0.165016	0.032525	0.124211	0.070575	0.092989	0.028615	0.022388
GLZLM_SZE	0.666951	0.763975	0.732865	0.718721	0.628402	0.779536	0.844692
GLZLM_LZE	8.719165	3.512315	4.367784	5.107981	10.006	2.984375	2.2875
GLZLM_LGZE	0.003039	0.004016	0.003556	0.002742	0.001751	0.002714	0.009606
GLZLM_HGZE	1354.156	1431.99	1078.554	1298.277	1644.673	1878.65	1775.738
GLZLM_SZLGE	0.00181	0.003706	0.002209	0.00247	0.001404	0.002555	0.009486
GLZLM_SZHGE	848.5225	1073.733	812.5203	927.3932	1013.009	1435.526	1515.977
GLZLM_LZLGE	0.01777	0.006777	0.017044	0.006765	0.008459	0.004193	0.01046
GLZLM_LZHGE	14543.41	5213.089	3701.87	6284.058	16748.85	5903.066	4081.056
GLZLM_GLNU	36.30044	9.990148	24.48231	24.64906	36.78111	16.25938	3.975
GLZLM_ZLNU	653.7122	219.6108	534.2328	407.8298	493.5697	361.0813	107.0875
GLZLM_ZP	0.496389	0.656958	0.617596	0.587586	0.462231	0.691145	0.769231

FLAIR							
Patient	15	16	17	18	19	20	21
Grade (II=0,III=1)	0	0	0	0	0	0	0
minValue	294.0274	440.8257	158.3439	523.4993	737.4102	451.3636	576.0248
meanValue	374.7344	521.299	220.3462	566.6474	786.0686	551.612	669.5126
stdValue	25.70697	30.41651	19.59409	16.9265	15.93331	25.70443	29.84937
maxValue	425.8351	600.5074	334.0182	618.4218	819.8351	613.2009	745.6286
HISTO_Skewness	-0.71256	0.10846	1.464242	-0.04153	-0.90985	-0.48499	-0.39444
HISTO_Kurtosis	3.081852	2.414506	7.793813	2.897214	3.805654	3.896699	3.089595
HISTO_Entropy_log10	1.671768	1.683014	1.406696	1.603716	1.518949	1.58257	1.637573
HISTO_Energy	0.025175	0.023209	0.051515	0.02895	0.036425	0.030612	0.027443
SHAPE_Volume	2.857633	3.75478	0.539286	1.471299	1.006224	4.818583	3.214205
GLCM_Homogeneity	0.204947	0.271559	0.36146	0.196881	0.153069	0.238246	0.200699
GLCM_Energy	0.00162	0.001946	0.004872	0.007331	0.01321	0.003074	0.006996
GLCM_Contrast	146.9321	48.80422	34.25334	144.3691	242.6998	74.33494	105.1267
GLCM_Correlation	0.529255	0.799569	0.692859	0.431212	0.298042	0.548785	0.276383
GLCM_Entropy_log10	2.898417	2.800943	2.55019	2.183422	1.911958	2.579941	2.251402
GLCM_Dissimilarity	9.377155	5.366635	3.844477	9.29554	11.96967	6.634231	8.062891
GLRLM_SRE	0.973733	0.965186	0.937749	0.982677	0.990946	0.972619	0.989385
GLRLM_LRE	1.143421	1.180464	1.306456	1.069292	1.036217	1.120336	1.043337
GLRLM_LGRE	0.002094	0.00305	0.003116	0.009185	0.012377	0.004719	0.004692
GLRLM_HGRE	1726.597	1224.191	591.6872	1008.326	1613.41	1711.605	1403.083
GLRLM_SRLGE	0.002049	0.002987	0.002967	0.009153	0.012371	0.004598	0.004684
GLRLM_SRHGE	1678.886	1182.864	560.3437	992.1635	1596.873	1663.394	1386.201
GLRLM_LRLGE	0.002307	0.003369	0.003849	0.009317	0.012403	0.005213	0.004728
GLRLM_LRHGE	1988.338	1433.684	744.3314	1072.975	1679.555	1923.82	1471.624
GLRLM_GLNU	51.4515	30.99667	118.8662	5.616369	3.571503	17.79971	10.74256
GLRLM_RLNU	1934.751	1235.247	2027.766	186.9313	95.62112	545.3316	382.8749
GLRLM_RP	0.963377	0.951909	0.915944	0.977692	0.988345	0.96395	0.986119
NGLDM_Coarseness	0.004341	0.010227	0.004693	0.032782	0.04505	0	0.022163
NGLDM_Contrast	0.389738	0.236872	0.057537	0.670241	1.385817	0	0.339317
NGLDM_Busyness	0.089063	0.054454	0.112036	0.023841	0.015194	0	0.020717
GLZLM_SZE	0.735377	0.690008	0.66351	0.806383	0.88366	0.721227	0.870008
GLZLM_LZE	4.508839	7.041835	55.2593	2.15894	1.517647	3.989276	1.736364
GLZLM_LGZE	0.002411	0.004016	0.00426	0.011289	0.014131	0.00453	0.005432
GLZLM_HGZE	1691.236	1246.829	686.3433	1036.642	1592.129	1692.74	1370.3
GLZLM_SZLGE	0.001979	0.00328	0.003414	0.010863	0.014041	0.002322	0.005319
GLZLM_SZHGE	1228.062	860.5407	483.9366	861.3133	1394.088	1212.309	1166.648
GLZLM_LZLGE	0.007152	0.013552	0.13268	0.01352	0.014509	0.014582	0.006032
GLZLM_LZHGE	8219.07	8190.594	25181.66	2099.861	2525.682	6959.279	2535.748
GLZLM_GLNU	30.49577	15.80432	33.45696	3.980132	3.188235	10.64075	8.484848
GLZLM_ZLNU	650.837	326.5547	383.3507	91.34437	62.69412	180.3351	235.2848
GLZLM_ZP	0.609653	0.524045	0.362062	0.755	0.858586	0.615512	0.827068

FLAIR							
Patient	22	23	24	25	26	27	28
Grade (II=0,III=1)	0	0	0	0	0	0	0
minValue	297.4063	321.6748	447.0511	522.5706	4195.889	384.8094	429.7038
meanValue	596.6879	508.1287	505.6154	589.8254	4709.177	664.0497	566.7184
stdValue	94.54043	37.50449	22.02213	27.28142	307.6221	113.5098	30.7688
maxValue	804.9855	562.225	573.0741	668.6502	5370.443	1045.402	636.8102
HISTO_Skewness	-0.20616	-2.18341	0.294936	-0.13627	0.375217	0.784211	-1.55145
HISTO_Kurtosis	2.616975	9.010772	3.394877	2.675612	2.023715	3.220903	6.734017
HISTO_Entropy_log10	1.671394	1.465123	1.643487	1.676738	1.620095	1.608419	1.518327
HISTO_Energy	0.024209	0.044879	0.02807	0.024046	0.027348	0.030312	0.038521
SHAPE_Volume	4.07047	2.319643	0.939593	1.761763	0.99831	2.447204	1.582493
GLCM_Homogeneity	0.207437	0.310454	0.197194	0.202025	0.136118	0.343331	0.246929
GLCM_Energy	0.001752	0.005796	0.002203	0.001821	0.010548	0.003071	0.003074
GLCM_Contrast	193.1617	28.04497	191.3009	161.8258	367.7644	35.89304	239.7537
GLCM_Correlation	0.25095	0.469265	0.366706	0.458817	0.259791	0.874116	0.073253
GLCM_Entropy_log10	2.898612	2.356327	2.751454	2.816353	2.009801	2.725886	2.666657
GLCM_Dissimilarity	10.54613	4.135863	10.30228	9.64685	15.21379	4.222354	10.36827
GLRLM_SRE	0.971707	0.968703	0.981385	0.97903	0.98841	0.941915	0.961731
GLRLM_LRE	1.263798	1.174694	1.10778	1.11838	1.049554	1.337501	1.237528
GLRLM_LGRE	0.002399	0.002427	0.005633	0.006596	0.02142	0.003107	0.004963
GLRLM_HGRE	1599.029	2597.397	1042.988	1042.658	1098.381	892.6257	1920.875
GLRLM_SRLGE	0.002329	0.002371	0.005526	0.006417	0.020825	0.002948	0.004524
GLRLM_SRHGE	1551.157	2509.641	1025.497	1021.729	1088.581	848.8143	1844.713
GLRLM_LRLGE	0.002834	0.002667	0.006942	0.007524	0.023805	0.003977	0.008448
GLRLM_LRHGE	2056.131	3098.066	1145.642	1158.81	1140.652	1145.091	2398.281
GLRLM_GLNU	47.07371	63.86447	34.63889	28.70133	3.12664	73.3754	88.55067
GLRLM_RLNU	1847.888	1342.642	1191.421	1136.334	110.9941	2164.002	2144.148
GLRLM_RP	0.957785	0.956868	0.972957	0.969968	0.984748	0.919403	0.945894
NGLDM_Coarseness	0.003522	0.007277	0.006939	0.006282	0.032613	0.007322	0.003318
NGLDM_Contrast	0.485838	0.11973	0.323755	0.444699	2.383266	0.149012	0.190777
NGLDM_Busyness	0.126787	0.030798	0.076525	0.087845	0.026713	0.091334	0.083041
GLZLM_SZE	0.732624	0.713312	0.818059	0.780593	0.854459	0.580053	0.629964
GLZLM_LZE	6.660177	6.336482	3.168142	3.425455	1.747368	37.78333	9.860122
GLZLM_LGZE	0.002576	0.002727	0.00383	0.00601	0.015137	0.003584	0.002665
GLZLM_HGZE	1536.011	2446.769	1077.633	1063.452	1142.884	1019.756	1842.49
GLZLM_SZLGE	0.001606	0.001694	0.00233	0.004026	0.006546	0.001844	0.001224
GLZLM_SZHGE	1100.815	1663.734	899.8112	842.7233	1011.676	614.8495	1127.353
GLZLM_LZLGE	0.010428	0.008336	0.024905	0.023547	0.050093	0.095446	0.094759
GLZLM_LZHGE	11359.52	17718.92	3101.7	3456.833	1786.2	22051.02	19860.43
GLZLM_GLNU	26.29469	31.12515	23.05088	19.45091	2.621053	22.01556	36.71156
GLZLM_ZLNU	561.2575	398.6954	565.2765	466.5952	65.12632	283.5933	428.5152
GLZLM_ZP	0.554465	0.562044	0.70625	0.669643	0.818966	0.33321	0.467696

FLAIR							
Patient	29	30	31	32	33	34	35
Grade (II=0,III=1)	0	0	0	0	0	0	0
minValue	236.6749	385.8445	3387.721	220.7032	126.6309	368.7443	523.7197
meanValue	268.0693	638.9472	4970.315	284.8767	151.5503	463.4854	611.7992
stdValue	15.2152	51.69023	395.0778	29.39777	7.550942	37.60358	30.81551
maxValue	311.5824	720.4316	5977.929	366.8092	170.4166	575.1838	690.9822
HISTO_Skewness	0.509066	-1.84217	-0.39992	0.381632	-0.1831	0.617492	0.422964
HISTO_Kurtosis	2.989671	6.566729	4.272728	2.441663	2.628713	3.06088	2.843603
HISTO_Entropy_log10	1.692262	1.444639	1.524822	1.692888	1.641058	1.650427	1.649097
HISTO_Energy	0.023484	0.051582	0.036532	0.022785	0.02589	0.026931	0.0269
SHAPE_Volume	1.560026	1.797917	2.775223	3.725149	0.222791	6.561372	3.864179
GLCM_Homogeneity	0.205254	0.368928	0.241982	0.274322	0.200438	0.326902	0.226037
GLCM_Energy	0.002776	0.006903	0.004844	0.001702	0.001649	0.003312	0.002305
GLCM_Contrast	134.9067	29.5738	84.13232	89.5363	108.8777	67.88449	169.3111
GLCM_Correlation	0.488152	0.762201	0.448565	0.69666	0.542106	0.661124	0.245774
GLCM_Entropy_log10	2.626509	2.379842	2.377779	2.891075	2.85102	2.775146	2.738393
GLCM_Dissimilarity	8.952665	3.601736	6.944406	6.557821	8.206737	5.883843	9.145114
GLRLM_SRE	0.981427	0.940151	0.975742	0.958711	0.980657	0.912009	0.975681
GLRLM_LRE	1.104913	1.302701	1.106308	1.208761	1.080173	2.566843	1.132917
GLRLM_LGRE	0.010205	0.001489	0.003886	0.004001	0.002439	0.002506	0.002593
GLRLM_HGRE	913.3105	2464.982	1662.384	987.4109	1485.877	1033.468	1309.641
GLRLM_SRLGE	0.009961	0.001464	0.003868	0.003861	0.002421	0.002296	0.002566
GLRLM_SRHGE	895.419	2299.293	1621.347	948.0999	1456.894	944.722	1278.619
GLRLM_LRLGE	0.01127	0.001614	0.003961	0.004676	0.002514	0.00565	0.002741
GLRLM_LRHGE	1017.272	3308.257	1842.719	1183.512	1606.4	2581.495	1480.727
GLRLM_GLNU	17.65519	65.76395	13.10028	44.58424	24.04688	453.3033	30.37559
GLRLM_RLNU	721.7138	1151.521	340.6182	1790.516	885.2864	15116.16	1076.479
GLRLM_RP	0.973201	0.91917	0.96812	0.944598	0.974493	0.879239	0.965704
NGLDM_Coarseness	0.010935	0.00872	0.018222	0.005563	0.008532	0.000603	0.006826
NGLDM_Contrast	0.456663	0.114737	0.371837	0.341118	0.369172	0.229085	0.413064
NGLDM_Busyness	0.065146	0.026663	0.026338	0.128261	0.056073	1.016103	0.078575
GLZLM_SZE	0.801822	0.667509	0.760662	0.638471	0.806278	0.385245	0.759829
GLZLM_LZE	3.018349	41.15665	3.428571	10.49446	2.760933	307.2866	4.509485
GLZLM_LGZE	0.009327	0.002965	0.005389	0.004312	0.00297	0.003209	0.003398
GLZLM_HGZE	898.655	2116.863	1662.69	1018.901	1478.28	1030.235	1318.436
GLZLM_SZLGE	0.005672	0.002739	0.005196	0.002479	0.002781	0.001333	0.003132
GLZLM_SZHGE	714.9383	1311.932	1288.826	655.2891	1183.041	399.8403	997.3752
GLZLM_LZLGE	0.026416	0.017364	0.007029	0.031841	0.004637	0.494761	0.007393
GLZLM_LZHGE	2876.921	118537.6	5972.555	9265.74	4081.417	294377.9	5538.21
GLZLM_GLNU	12.28807	15.64481	7.75102	21.07654	17.30904	62.91654	17.51762
GLZLM_ZLNU	326.0459	227	131.4408	379.7513	415.1108	444.6091	394.8022
GLZLM_ZP	0.703226	0.376802	0.65508	0.474892	0.717573	0.140963	0.624365

FLAIR							
Patient	36	37	38	39	40	41	42
Grade (II=0,III=1)	0	0	0	0	0	0	0
minValue	193.3544	287.1258	440.304	524.3306	4320.024	379.6324	335.1268
meanValue	292.8752	516.2752	689.1361	562.1789	6786.067	427.9841	429.4693
stdValue	31.51351	83.71288	47.16272	12.12594	1254.646	18.88579	27.37856
maxValue	442.6342	681.4423	786.7017	588.5028	9025.097	485.6592	486.6553
HISTO_Skewness	-0.29354	-0.38436	-1.02125	-0.3128	0.131347	0.273304	-0.46201
HISTO_Kurtosis	3.583019	2.416533	5.092253	2.842014	1.820471	3.027778	2.929085
HISTO_Entropy_log10	1.496308	1.695553	1.509066	1.648633	1.672118	1.649144	1.657544
HISTO_Energy	0.038141	0.022261	0.037723	0.025744	0.025686	0.026795	0.025
SHAPE_Volume	4.145843	7.94826	3.437503	2.477583	2.141009	4.968909	3.28772
GLCM_Homogeneity	0.248328	0.180468	0.275036	0.180101	0.172428	0.18226	0.228637
GLCM_Energy	0.003258	0.002028	0.004583	0.004878	0.01318	0.0017	0.00171
GLCM_Contrast	74.34417	203.244	65.73341	140.0501	290.8018	199.5678	98.83974
GLCM_Correlation	0.509412	0.423856	0.20392	0.453202	0.381314	0.289184	0.639912
GLCM_Entropy_log10	2.602138	2.741382	2.443624	2.362287	2.009091	2.853949	2.879981
GLCM_Dissimilarity	6.589479	10.99408	5.865144	9.555156	12.5802	10.93551	7.487986
GLRLM_SRE	0.968375	0.978999	0.970824	0.985756	0.986439	0.97843	0.973256
GLRLM_LRE	1.164088	1.089392	1.139617	1.059369	1.063756	1.102527	1.169052
GLRLM_LGRE	0.005384	0.006668	0.001881	0.004607	0.009923	0.005409	0.002484
GLRLM_HGRE	742.6741	1602.416	2230.311	1612.574	1446.125	1012.154	1754.724
GLRLM_SRLGE	0.005254	0.006529	0.001867	0.004592	0.009908	0.00528	0.002423
GLRLM_SRHGE	718.4407	1566.263	2162.053	1591.158	1421.712	991.4048	1704.142
GLRLM_LRLGE	0.005977	0.007228	0.001946	0.00467	0.009999	0.006322	0.002974
GLRLM_LRHGE	870.0986	1759.703	2559.489	1700.866	1552.841	1114.714	2083.51
GLRLM_GLNU	44.2207	12.84162	29.73036	7.600641	4.465051	30.82324	50.87597
GLRLM_RLNU	1082.597	548.8195	742.0778	285.5372	169.9812	1095.686	1921.489
GLRLM_RP	0.957737	0.972592	0.960843	0.981151	0.981521	0.970952	0.961303
NGLDM_Coarseness	0.007399	0.011647	0.010253	0.023104	0.026398	0.005122	0.004947
NGLDM_Contrast	0.187936	0.764561	0.210159	0.583716	1.539592	0.474748	0.292131
NGLDM_Busyness	0.093365	0.044674	0.035434	0.023146	0.020118	0.110801	0.082934
GLZLM_SZE	0.708868	0.770533	0.718671	0.840745	0.849377	0.737398	0.734072
GLZLM_LZE	5.485549	2.827338	4.781633	2.162393	2.092199	3.050063	5.387149
GLZLM_LGZE	0.006229	0.006517	0.002752	0.005564	0.012066	0.004092	0.002126
GLZLM_HGZE	729.9668	1559.424	2167.624	1628.697	1376.298	1013.121	1664.79
GLZLM_SZLGE	0.004484	0.004428	0.00261	0.005407	0.011912	0.002183	0.001183
GLZLM_SZHGE	516.7596	1206.329	1533.34	1368.517	1088.637	782.1067	1190.784
GLZLM_LZLGE	0.019914	0.016094	0.004477	0.006763	0.013536	0.019947	0.011434
GLZLM_LZHGE	4363.497	4858.403	11244.38	3337.526	3042.227	3031.914	10532.39
GLZLM_GLNU	25.82659	8.81295	16.31429	5.717949	3.170213	19.31539	29.52691
GLZLM_ZLNU	321.7457	229.9688	234.6612	154.906	95.42553	402.8248	620.6145
GLZLM_ZP	0.570016	0.705584	0.591074	0.774834	0.787709	0.6966	0.585882

FLAIR							
Patient	43	44	45	46	47	48	49
Grade (II=0,III=1)	0	0	1	1	1	1	1
minValue	505.8029	663.6478	539.4081	2991.277	186.7985	465.786	3070.942
meanValue	633.3136	833.3217	727.5449	4487.624	265.1774	595.4535	5269.764
stdValue	28.11671	96.28456	51.5452	455.7809	23.79614	47.11783	499.9288
maxValue	692.9777	1072.235	882.567	5492.25	337.4449	694.7026	6736.523
HISTO_Skewness	-0.67912	0.32303	-0.32101	-0.3791	-0.18951	-0.31831	-0.46892
HISTO_Kurtosis	3.743026	2.292022	3.521308	2.935968	3.296855	2.366665	3.705184
HISTO_Entropy_log10	1.568599	1.723087	1.581618	1.661477	1.608798	1.710437	1.532717
HISTO_Energy	0.031867	0.021182	0.031958	0.024747	0.030153	0.021525	0.03433
SHAPE_Volume	1.423649	0.500462	2.817326	19.7499	2.765549	2.444306	8.076387
GLCM_Homogeneity	0.241886	0.189823	0.246231	0.185508	0.329024	0.248094	0.269563
GLCM_Energy	0.002645	0.007038	0.002071	0.001607	0.002464	0.002086	0.002455
GLCM_Contrast	151.1063	209.7637	68.92517	207.848	36.22181	153.8652	63.13696
GLCM_Correlation	0.143192	0.603326	0.588201	0.196166	0.818854	0.190157	0.613067
GLCM_Entropy_log10	2.725221	2.23983	2.79212	2.864544	2.808141	2.881021	2.714632
GLCM_Dissimilarity	8.919308	10.87859	6.346601	10.75017	4.450916	9.100649	5.813752
GLRLM_SRE	0.96031	0.98566	0.970651	0.980874	0.947248	0.942148	0.963177
GLRLM_LRE	1.260397	1.06597	1.13472	1.090739	1.349458	2.022511	1.169941
GLRLM_LGRE	0.001594	0.019599	0.002022	0.002647	0.002194	0.00256	0.002643
GLRLM_HGRE	2028.8	963.6992	1356.741	1641.293	1254.898	1511.104	1587.573
GLRLM_SRLGE	0.001538	0.018951	0.00199	0.002627	0.002038	0.00244	0.002552
GLRLM_SRHGE	1944.063	951.6019	1316.055	1610.293	1194.815	1419.203	1528.093
GLRLM_LRLGE	0.001869	0.022218	0.002164	0.002739	0.003121	0.004862	0.003026
GLRLM_LRHGE	2599.647	1017.499	1544.352	1786.505	1645.968	3226.088	1857.025
GLRLM_GLNU	71.01238	6.579984	44.11631	27.17179	258.3822	167.7475	36.31022
GLRLM_RLNU	2067.302	300.8589	1292.972	1052.902	7669.138	7274.635	969.6656
GLRLM_RP	0.944159	0.980406	0.96069	0.973997	0.924714	0.928433	0.951381
NGLDM_Coarseness	0.0035	0.02759	0.006693	0.005965	0.001993	0.001437	0.008738
NGLDM_Contrast	0.234079	0.70301	0.203584	0.514418	0.102311	0.339529	0.184636
NGLDM_Busyness	0.098367	0.031528	0.065403	0.071062	0.216445	0.327356	0.050512
GLZLM_SZE	0.658364	0.840879	0.712976	0.793511	0.563093	0.42294	0.690965
GLZLM_LZE	11.68643	2.154472	4.976471	2.998731	29.38395	29.31478	7.192833
GLZLM_LGZE	0.001809	0.012203	0.002601	0.003286	0.002029	0.002597	0.002582
GLZLM_HGZE	1919.164	999.3659	1336.467	1649.718	1380.364	1360.53	1582.162
GLZLM_SZLGE	0.000923	0.007593	0.002229	0.003035	0.001033	0.001026	0.001037
GLZLM_SZHGE	1218.496	848.7221	937.9619	1311.707	807.1355	503.8031	1095.34
GLZLM_LZLGE	0.010183	0.079027	0.006486	0.005163	0.054677	0.055311	0.012315
GLZLM_LZHGE	25217.79	1877.679	6871.619	4858.096	30728.9	49556.24	11593.12
GLZLM_GLNU	32.54358	5.056911	24.31765	18.22335	90.07673	50.38343	18.88737
GLZLM_ZLNU	447.7637	162.9187	400.0706	461.2259	943.8074	515.3046	258.7986
GLZLM_ZP	0.467844	0.773585	0.586207	0.695499	0.33756	0.292018	0.52462

FLAIR							
Patient	50	51	52	53	54	55	56
Grade (II=0,III=1)	1	1	1	1	1	1	1
minValue	4690.442	137.4663	3627.4	457.4416	4160.473	351.2564	4247.353
meanValue	6698.983	201.1767	5971.841	568.619	5313.77	416.5948	5880.497
stdValue	798.4061	11.96077	702.9754	21.43042	454.3027	23.229	336.9671
maxValue	8695.229	232.8693	8277.752	620.9244	7743.471	479.2495	6596.398
HISTO_Skewness	0.377116	-1.07737	0.356834	-0.82532	1.432052	0.136935	-1.6014
HISTO_Kurtosis	3.099116	5.6102	3.109213	4.63915	6.423616	2.143304	6.372569
HISTO_Entropy_log10	1.501134	1.480447	1.58116	1.508604	1.457014	1.646032	1.48605
HISTO_Energy	0.036898	0.040367	0.031504	0.036616	0.044155	0.025437	0.041628
SHAPE_Volume	1.442048	0.492155	18.92935	1.801577	14.5783	11.18177	5.446668
GLCM_Homogeneity	0.122496	0.30799	0.255074	0.263331	0.26926	0.236808	0.275292
GLCM_Energy	0.09613	0.003032	0.002015	0.002675	0.003137	0.001453	0.003025
GLCM_Contrast	430.6003	39.1634	72.12224	57.88537	53.86322	82.83136	65.89241
GLCM_Correlation	-0.13519	0.653994	0.61739	0.526431	0.418715	0.673608	0.474104
GLCM_Entropy_log10	1.34736	2.666713	2.81252	2.680447	2.646896	2.967245	2.66435
GLCM_Dissimilarity	16.62504	4.527926	6.321127	5.771386	5.552917	6.940401	5.782073
GLRLM_SRE	0.990919	0.955158	0.969083	0.962408	0.964397	0.971548	0.962364
GLRLM_LRE	1.040042	1.212579	1.136398	1.176662	1.16684	1.142261	1.171913
GLRLM_LGRE	0.012228	0.001522	0.00211	0.001701	0.004035	0.001945	0.00148
GLRLM_HGRE	1224.353	1926.967	1167.569	2003.898	516.4282	1235.513	2097.487
GLRLM_SRLGE	0.012213	0.001497	0.002075	0.001681	0.003909	0.001908	0.001461
GLRLM_SRHGE	1213.885	1835.695	1131.825	1924.643	501.8683	1200.39	2010.376
GLRLM_LRLGE	0.01229	0.001639	0.002263	0.001795	0.004622	0.002128	0.001567
GLRLM_LRHGE	1269.139	2362.853	1325.325	2379.93	583.915	1410.379	2495.145
GLRLM_GLNU	3.44536	104.3068	50.24101	44.55709	83.41406	78.55129	64.96556
GLRLM_RLNU	91.80466	2331.545	1484.762	1118.102	1752.729	2899.076	1441.269
GLRLM_RP	0.987854	0.939483	0.959538	0.950188	0.952454	0.960778	0.949839
NGLDM_Coarseness	0.05099	0.003525	0.005918	0.006855	0.003705	0.003015	0.005039
NGLDM_Contrast	1.500348	0.108718	0.206137	0.178725	0.146905	0.321939	0.184164
NGLDM_Busyness	0.020828	0.084039	0.085917	0.053127	0.192251	0.178142	0.057593
GLZLM_SZE	0.875781	0.687567	0.707467	0.669287	0.681481	0.731589	0.697528
GLZLM_LZE	1.6375	17.36938	4.943416	6.313697	7.241769	4.935818	8.159302
GLZLM_LGZE	0.014069	0.00252	0.002742	0.00263	0.004463	0.002319	0.002285
GLZLM_HGZE	1238.625	1851.334	1174.948	1925.029	593.3942	1249.066	1942.387
GLZLM_SZLGE	0.013871	0.002307	0.002399	0.002441	0.003372	0.001942	0.002101
GLZLM_SZHGE	1091.044	1253.527	837.5024	1247.6	442.4426	916.1237	1287.151
GLZLM_LZLGE	0.01504	0.010665	0.007137	0.005393	0.027539	0.007376	0.00562
GLZLM_LZHGE	1939.2	37239.88	5631.19	13380	2834.306	5914.735	18913.59
GLZLM_GLNU	2.75	42.73796	27.57922	22.53166	38.85042	44.88923	28.37558
GLZLM_ZLNU	57.925	553.7167	451.4743	282.4963	457.4111	956.4772	386.7593
GLZLM_ZP	0.842105	0.454937	0.581688	0.525949	0.528856	0.596665	0.515897

FLAIR							
Patient	57	58	59	60	61	62	63
Grade (II=0,III=1)	1	1	1	1	1	1	1
minValue	478.0447	470.8609	364.8465	605.4285	542.3687	446.0406	228
meanValue	612.2058	748.5666	613.4676	780.025	758.4826	603.86	590.8765
stdValue	48.81224	57.13023	56.3591	75.86159	50.67623	41.36555	92.36596
maxValue	717.7327	951.672	751.678	941.2985	904.7761	712.5391	927
HISTO_Skewness	-0.35445	-0.4648	-0.87437	-0.37882	-0.35385	-0.13803	1.056795
HISTO_Kurtosis	2.592513	4.716992	4.09904	2.48573	4.491313	2.747631	4.506141
HISTO_Entropy_log10	1.700819	1.479875	1.552704	1.705167	1.537986	1.603115	1.446096
HISTO_Energy	0.022602	0.040859	0.033436	0.023766	0.035709	0.028201	0.049727
SHAPE_Volume	1.693455	3.812878	8.305322	3.684389	0.609374	4.941036	12.12081
GLCM_Homogeneity	0.202335	0.276006	0.247644	0.221817	0.257751	0.271987	0.333128
GLCM_Energy	0.002478	0.002842	0.001864	0.002838	0.003334	0.003041	0.005239
GLCM_Contrast	134.7388	84.62409	86.45044	111.1872	102.1635	79.65328	85.04694
GLCM_Correlation	0.568218	0.254755	0.513186	0.735615	0.35353	0.637183	0.277687
GLCM_Entropy_log10	2.667839	2.720714	2.869752	2.605125	2.570761	2.905252	2.537029
GLCM_Dissimilarity	9.01983	6.360344	6.766616	7.971731	7.040018	6.876056	5.657774
GLRLM_SRE	0.974525	0.959409	0.969028	0.976377	0.968128	0.906568	0.939872
GLRLM_LRE	1.132165	1.217637	1.14225	1.10666	1.148581	23.25003	1.298397
GLRLM_LGRE	0.005128	0.001438	0.001956	0.010183	0.002492	0.001224	0.001746
GLRLM_HGRE	1484.708	1460.198	1813.62	1342.051	1575.108	1564.056	1219.641
GLRLM_SRLGE	0.005086	0.001397	0.001909	0.010096	0.002469	0.001105	0.001685
GLRLM_SRHGE	1443.752	1400.297	1752.937	1305.692	1524.691	1425.828	1153.867
GLRLM_LRLGE	0.005325	0.00164	0.00215	0.010547	0.002598	0.02247	0.00205
GLRLM_LRHGE	1702.038	1786.052	2095.845	1505.564	1809.849	39086.37	1545.746
GLRLM_GLNU	17.80746	145.954	91.29567	11.46953	26.06247	1958.853	86.12268
GLRLM_RLNU	747.2106	3292.909	2542.449	459.2528	683.6012	65683.46	1560.851
GLRLM_RP	0.965239	0.944501	0.958801	0.968497	0.957553	0.903795	0.920261
NGLDM_Coarseness	0.011723	0.002369	0.002809	0.01845	0.010435	0.00014	0.003303
NGLDM_Contrast	0.421865	0.098844	0.204894	0.483847	0.216102	0.166152	0.185381
NGLDM_Busyness	0.039278	0.133302	0.112961	0.025308	0.038626	2.7584	0.165727
GLZLM_SZE	0.738709	0.662794	0.706294	0.762559	0.719545	0.035087	0.679852
GLZLM_LZE	3.899225	10.07898	5.489336	3.547112	5.291196	629.0158	52.96216
GLZLM_LGZE	0.006911	0.001803	0.002216	0.013271	0.003663	0.001427	0.002785
GLZLM_HGZE	1415.543	1444.545	1731.782	1252.027	1578.932	1455.068	1359.519
GLZLM_SZLGE	0.006388	0.001336	0.001497	0.012102	0.003452	5.05E-05	0.00247
GLZLM_SZHGE	1020.694	952.9612	1191.609	902.5669	1130.978	46.39756	948.1529
GLZLM_LZLGE	0.011344	0.009357	0.006855	0.020643	0.006691	0.55107	0.055049
GLZLM_LZHGE	6244.8	15287.08	10948.45	5337.027	8360.332	1077399	54851.96
GLZLM_GLNU	11.05233	63.63508	50.43327	7.170213	13.6456	143.8464	22.52297
GLZLM_ZLNU	260.905	749.61	758.61	177.2188	212.4447	253.4103	316.6581
GLZLM_ZP	0.629268	0.479248	0.572575	0.654076	0.573834	0.066583	0.376399

FLAIR							
Patient	64	65	66	67	68	69	70
Grade (II=0,III=1)	1	1	1	1	1	1	1
minValue	2401	1046	330	115	1	46	2285
meanValue	4576.704	3244.49	709.855	286.8199	100.7575	466.4176	2825.833
stdValue	520.2054	1111.523	207.1056	39.85975	41.91242	37.15192	175.4715
maxValue	7558	6949	1826	451	306	645	3483
HISTO_Skewness	-0.04471	1.315974	1.846536	-0.39518	0.065634	-0.12956	0.137089
HISTO_Kurtosis	4.434947	3.969517	7.688978	4.879961	3.598416	8.099681	2.858308
HISTO_Entropy_log10	1.40832	1.556656	1.419266	1.437428	1.535843	1.189367	1.584371
HISTO_Energy	0.05044	0.038829	0.050867	0.053202	0.034763	0.075665	0.029882
SHAPE_Volume	25.40809	16.21677	38.33811	14.65782	25.61773	51.29899	14.03572
GLCM_Homogeneity	0.325922	0.313952	0.405113	0.356012	0.276637	0.578227	0.22297
GLCM_Energy	0.003953	0.003612	0.009438	0.006409	0.002176	0.017253	0.001323
GLCM_Contrast	42.16098	66.07589	66.00649	69.97367	99.0065	4.95919	101.1013
GLCM_Correlation	0.478314	0.70731	0.551135	0.308972	0.433236	0.794411	0.343504
GLCM_Entropy_log10	2.633877	2.714084	2.50053	2.597372	2.902208	1.972331	3.002626
GLCM_Dissimilarity	4.549696	5.429432	5.07946	5.391503	7.118388	1.401999	7.652016
GLRLM_SRE	0.948968	0.945527	0.882655	0.91968	0.94787	0.8052	0.973087
GLRLM_LRE	1.260052	1.285842	2.677237	1.566438	1.317169	3.412593	1.119298
GLRLM_LGRE	0.001991	0.004113	0.006641	0.001687	0.01335	0.000547	0.002178
GLRLM_HGRE	799.2389	748.8694	398.8852	1161.377	538.1007	2083.519	953.0238
GLRLM_SRLGE	0.001912	0.003943	0.005619	0.001581	0.012319	0.000451	0.002128
GLRLM_SRHGE	759.2692	715.9273	370.0994	1067.313	511.103	1677.767	928.2822
GLRLM_LRLGE	0.002389	0.005017	0.022484	0.002335	0.020181	0.001751	0.002398
GLRLM_LRHGE	1003.484	917.9493	718.4797	1829.373	704.0983	7025.037	1062.032
GLRLM_GLNU	358.9668	180.2075	1040.409	466.1544	574.9261	2063.278	275.3194
GLRLM_RLNU	6441.249	4215.816	18111.13	7854.406	14867.43	19078.27	8649.434
GLRLM_RP	0.932192	0.927427	0.821674	0.888163	0.928391	0.748055	0.96418
NGLDM_Coarseness	0.001262	0.002062	0.000239	0.000662	0.000509	0	0.000828
NGLDM_Contrast	0.061054	0.234068	0.139826	0.103424	0.146693	0	0.219692
NGLDM_Busyness	0.345487	0.33481	3.341487	0.496938	1.153252	0	0.602029
GLZLM_SZE	0.642689	0.65871	0.609882	0.616367	0.617928	0.49547	0.743507
GLZLM_LZE	27.87089	42.23134	3319.478	419.6338	25.38194	28022.11	4.34851
GLZLM_LGZE	0.002517	0.005514	0.004002	0.002373	0.010745	0.001088	0.002324
GLZLM_HGZE	817.2726	873.5052	687.7724	1111.824	549.6182	2110.376	971.6517
GLZLM_SZLGE	0.001901	0.004472	0.002165	0.001505	0.006463	0.000827	0.001766
GLZLM_SZHGE	534.0258	594.8625	471.0031	660.1856	344.8558	1030.513	730.9688
GLZLM_LZLGE	0.039907	0.140019	36.49394	0.381581	1.547373	13.77068	0.008201
GLZLM_LZHGE	21834.12	17351.92	341593.6	472967.1	10511.24	57886763	3948.459
GLZLM_GLNU	124.8659	53.75927	139.1746	86.31045	224.1007	107.2077	165.6481
GLZLM_ZLNU	1225.129	812.5393	1603.349	1045.177	2503.363	544.1675	3057.043
GLZLM_ZP	0.409207	0.391751	0.168732	0.280311	0.391896	0.06105	0.621774

FLAIR							
Patient	71	72	73	74	75	76	77
Grade (II=0,III=1)	1	1	1	1	1	1	1
minValue	239	28	67	180	698	1002	69
meanValue	499.2813	416.9464	593.3107	408.8764	5108.424	5212.621	401.805
stdValue	56.37394	73.91664	93.3187	46.71339	962.8636	874.5792	83.03336
maxValue	647	670	849	598	7328	6725	655
HISTO_Skewness	-0.29462	0.450608	-0.9163	-0.12283	-0.72499	-0.70294	-0.42974
HISTO_Kurtosis	3.078673	3.146759	3.84489	4.631398	3.437049	3.429534	3.549623
HISTO_Entropy_log10	1.549733	1.460448	1.455047	1.44924	1.550178	1.518276	1.55965
HISTO_Energy	0.032218	0.041776	0.043217	0.046205	0.034673	0.037121	0.033602
SHAPE_Volume	19.89536	27.37898	20.1029	41.23384	42.96912	46.69097	21.47739
GLCM_Homogeneity	0.303013	0.364711	0.350635	0.33215	0.31044	0.341637	0.314892
GLCM_Energy	0.002947	0.004427	0.004428	0.003811	0.002577	0.00408	0.002985
GLCM_Contrast	60.44495	39.12479	40.94635	44.20213	80.78252	68.32866	91.72383
GLCM_Correlation	0.542001	0.624644	0.587475	0.539843	0.560782	0.650328	0.454056
GLCM_Entropy_log10	2.769793	2.630485	2.623167	2.69339	2.871955	2.766915	2.836927
GLCM_Dissimilarity	5.705235	4.195034	4.40326	4.633848	5.965075	5.392579	6.51047
GLRLM_SRE	0.9317	0.914405	0.930921	0.932647	0.940251	0.92959	0.935112
GLRLM_LRE	1.549458	1.609616	1.48473	1.376595	1.324418	1.404382	1.575558
GLRLM_LGRE	0.00091	0.000793	0.000768	0.001146	0.000764	0.00072	0.001536
GLRLM_HGRE	1780.51	1603.037	1938.685	1314.157	1931.736	2310.687	1426.765
GLRLM_SRLGE	0.000858	0.000731	0.000725	0.001081	0.000727	0.000691	0.001454
GLRLM_SRHGE	1654.381	1470.127	1794.007	1225.543	1809.648	2116.907	1329.023
GLRLM_LRLGE	0.001297	0.001236	0.001043	0.001504	0.00096	0.000881	0.002106
GLRLM_LRHGE	2787.02	2541.343	2968.2	1811.938	2598.91	3458.937	2337.539
GLRLM_GLNU	416.4347	680.2051	585.5805	1163.351	436.7429	361.7474	462.5494
GLRLM_RLNU	11426.86	14007.11	12002.58	22029.54	11192.29	8696.486	12316.97
GLRLM_RP	0.903942	0.886162	0.902083	0.910403	0.919469	0.902901	0.9035
NGLDM_Coarseness	0.000784	0.000624	0.000665	0.000305	0.000686	0.000825	0.000607
NGLDM_Contrast	0.125932	0.082909	0.08368	0.076675	0.1775	0.171412	0.152227
NGLDM_Busyness	0.44288	0.708272	0.442967	1.034903	0.529775	0.302668	0.562208
GLZLM_SZE	0.530883	0.509315	0.572377	0.577149	0.631816	0.668738	0.580184
GLZLM_LZE	76.34116	308.0989	144.8835	80.06358	52.95843	360.5517	146.2632
GLZLM_LGZE	0.001241	0.001023	0.000978	0.00139	0.000973	0.00114	0.002105
GLZLM_HGZE	1653.37	1667.315	1756.363	1301.604	1797.633	1911.946	1308.781
GLZLM_SZLGE	0.00064	0.000674	0.000517	0.000782	0.00071	0.000937	0.001294
GLZLM_SZHGE	832.1487	846.3617	970.2862	738.7244	1103.438	1226.297	744.2577
GLZLM_LZLGE	0.050175	0.238551	0.064876	0.069328	0.02863	0.109891	0.098401
GLZLM_LZHGE	138786.9	418429.1	340124.4	100144.6	111068.5	1209073	245592.8
GLZLM_GLNU	102.884	133.6885	147.7304	312.0552	141.3041	115.8113	130.4499
GLZLM_ZLNU	944.5819	990.4482	1371.974	2690.484	1837.881	1623.066	1398.385
GLZLM_ZP	0.245623	0.211489	0.285973	0.304043	0.353848	0.343025	0.286332

FLAIR							
Patient	78	79	80	81	82	83	84
Grade (II=0,III=1)	1	1	1	1	1	1	1
minValue	164	2788	490	1153	2577	346	192
meanValue	430.0206	4821.471	3835.179	2289.68	4622.376	613.1656	682.6508
stdValue	50.87103	471.618	1568.083	281.441	334.818	62.65038	79.47211
maxValue	598	6076	9903	4051	5961	823	928
HISTO_Skewness	-0.56716	-0.47636	1.20072	1.217659	0.000967	-0.74777	-1.12988
HISTO_Kurtosis	4.728242	3.417197	5.173198	6.234174	4.131582	3.625827	6.784453
HISTO_Entropy_log10	1.474318	1.564116	1.579662	1.366227	1.408861	1.504142	1.415623
HISTO_Energy	0.040822	0.03236	0.032671	0.055192	0.047038	0.038559	0.049493
SHAPE_Volume	28.69186	19.86157	33.82911	43.3734	9.150353	7.416979	25.03015
GLCM_Homogeneity	0.328017	0.267749	0.277617	0.34736	0.281575	0.269425	0.323934
GLCM_Energy	0.003523	0.00193	0.002231	0.005164	0.003178	0.002911	0.004096
GLCM_Contrast	42.07227	87.30944	114.2483	30.64913	41.56615	124.7184	58.4153
GLCM_Correlation	0.584511	0.448384	0.523289	0.505141	0.371307	0.153439	0.410396
GLCM_Entropy_log10	2.670587	2.903692	2.933469	2.523902	2.622344	2.779402	2.657382
GLCM_Dissimilarity	4.565852	6.598182	7.451412	3.996331	4.968277	7.998731	5.044497
GLRLM_SRE	0.935096	0.954771	0.944417	0.93037	0.961278	0.94881	0.939316
GLRLM_LRE	1.382448	1.215465	1.332559	1.410586	1.177647	1.333564	1.34962
GLRLM_LGRE	0.001227	0.001028	0.004294	0.001861	0.001041	0.001422	0.000752
GLRLM_HGRE	1628.648	1687.696	658.6946	700.4948	1578.606	1384.929	1904.488
GLRLM_SRLGE	0.001157	0.000996	0.00407	0.001733	0.001014	0.001364	0.000718
GLRLM_SRHGE	1519.017	1609.06	625.2945	655.7518	1519.754	1310.149	1783.622
GLRLM_LRLGE	0.001599	0.00118	0.005572	0.002615	0.001167	0.00175	0.000948
GLRLM_LRHGE	2276.639	2063.009	860.9207	961.1278	1847.98	1882.204	2606.108
GLRLM_GLNU	822.0982	183.4538	298.4835	1474.771	142.2554	188.2751	918.4322
GLRLM_RLNU	17660.33	5145.575	8227.393	23270.26	2770.805	4452.388	16430.74
GLRLM_RP	0.913556	0.940237	0.925113	0.906417	0.948344	0.927935	0.917441
NGLDM_Coarseness	0.000448	0.001319	0.000895	0.000265	0.002357	0.001154	0.000402
NGLDM_Contrast	0.073762	0.205258	0.239278	0.051386	0.102133	0.227752	0.075935
NGLDM_Busyness	0.648911	0.284113	0.728469	1.592717	0.253656	0.361761	0.627788
GLZLM_SZE	0.545974	0.661629	0.596032	0.56525	0.684671	0.623695	0.589984
GLZLM_LZE	48.15002	18.26993	29.68954	98.95968	10.31792	28.41931	47.0929
GLZLM_LGZE	0.001569	0.001359	0.004717	0.001943	0.001355	0.001721	0.001048
GLZLM_HGZE	1548.886	1639.463	708.3346	801.5089	1622.965	1304.265	1768.67
GLZLM_SZLGE	0.000842	0.001087	0.002901	0.001155	0.001132	0.000938	0.00075
GLZLM_SZHGE	818.2384	1057.956	450.4519	478.7743	1132.316	786.6955	991.6023
GLZLM_LZLGE	0.03447	0.01299	0.099428	0.182634	0.007936	0.023006	0.02472
GLZLM_LZHGE	83495.62	30023.98	17047.64	57629.62	15544.38	44166.45	96419.6
GLZLM_GLNU	227.1785	75.08925	95.43206	339.6366	63.35149	62.2896	250.3988
GLZLM_ZLNU	1870.389	1109.078	1207.452	2503.145	684.8334	730.9965	2154.433
GLZLM_ZP	0.296646	0.447463	0.369285	0.275381	0.490373	0.3775	0.321437

FLAIR					
Patient	85	86	87	88	89
Grade (II=0,III=1)	1	1	1	1	1
minValue	3048	243	216	144	65
meanValue	4375.316	552.0568	491.5389	300.1111	371.9095
stdValue	407.5302	92.07995	45.74656	58.18624	78.22227
maxValue	5928	796	621	463	560
HISTO_Skewness	-0.01071	0.051629	-0.43391	0.158381	-1.04103
HISTO_Kurtosis	3.243813	2.377525	3.94204	2.345306	4.253725
HISTO_Entropy_log10	1.56199	1.623193	1.460263	1.670315	1.564293
HISTO_Energy	0.032973	0.026524	0.042781	0.023463	0.036108
SHAPE_Volume	6.963797	18.22577	43.65422	15.13372	15.51917
GLCM_Homogeneity	0.229258	0.275494	0.307101	0.261689	0.329595
GLCM_Energy	0.001917	0.002045	0.003215	0.001579	0.003173
GLCM_Contrast	95.0658	99.56523	45.78643	179.657	55.66872
GLCM_Correlation	0.377237	0.513494	0.487105	0.330243	0.695072
GLCM_Entropy_log10	2.832105	2.918698	2.695092	3.066633	2.802831
GLCM_Dissimilarity	7.353221	7.271884	4.87253	9.538403	5.101625
GLRLM_SRE	0.97116	0.940917	0.950461	0.947484	0.93613
GLRLM_LRE	1.126237	1.469777	1.234636	1.475661	1.406657
GLRLM_LGRE	0.002424	0.001255	0.000734	0.002122	0.001997
GLRLM_HGRE	981.1019	1411.935	1984.245	1162.745	1703.747
GLRLM_SRLGE	0.002384	0.001191	0.000709	0.001981	0.001903
GLRLM_SRHGE	951.3658	1322.501	1881.242	1106.544	1587.528
GLRLM_LRLGE	0.002594	0.001709	0.000856	0.00342	0.002519
GLRLM_LRHGE	1111.369	2187.029	2472.961	1617.654	2457.342
GLRLM_GLNU	58.89832	319.5225	270.644	978.0603	440.2066
GLRLM_RLNU	1679.244	10708.93	5693.474	37559.46	10881.96
GLRLM_RP	0.962133	0.913826	0.934669	0.918933	0.911723
NGLDM_Coarseness	0.004369	0.000703	0.001327	0.000222	0.000887
NGLDM_Contrast	0.224521	0.267429	0.105816	0.320118	0.144052
NGLDM_Busyness	0.123769	0.671005	0.303197	2.521927	0.337016
GLZLM_SZE	0.73872	0.575218	0.648873	0.580579	0.589933
GLZLM_LZE	4.917326	65.47641	21.66504	53.67694	63.83434
GLZLM_LGZE	0.003019	0.00152	0.000993	0.001631	0.002681
GLZLM_HGZE	945.978	1257.52	1887.572	1306.738	1540.648
GLZLM_SZLGE	0.002596	0.000836	0.000797	0.000806	0.001638
GLZLM_SZHGE	672.1862	704.7394	1179.307	772.3517	863.9508
GLZLM_LZLGE	0.007969	0.046812	0.011302	0.158903	0.053086
GLZLM_LZHGE	4991.835	130598.7	45171.09	39576.23	121972.2
GLZLM_GLNU	32.9015	108.8932	99.84159	349.3046	114.7449
GLZLM_ZLNU	574.8716	1224.518	1119.197	4415.05	1402.321
GLZLM_ZP	0.605754	0.29959	0.416086	0.309275	0.31295
