# Supplementary material for: Ability of Radiomics in Differentiation of Anaplastic Oligodendroglioma From Atypical Low-Grade Oligodendroglioma Using Machine-Learning Approach
Source: Front Oncol. 2019 Dec 17;9:1371. doi: 10.3389/fonc.2019.01371 (PMC6929242; doi:10.3389/fonc.2019.01371)
Supplement: Supplementary Material 2 — Heat map showing Pearson's correlations between all pairs of features. [file Data_Sheet_2.pdf]

Supplementary Material 2

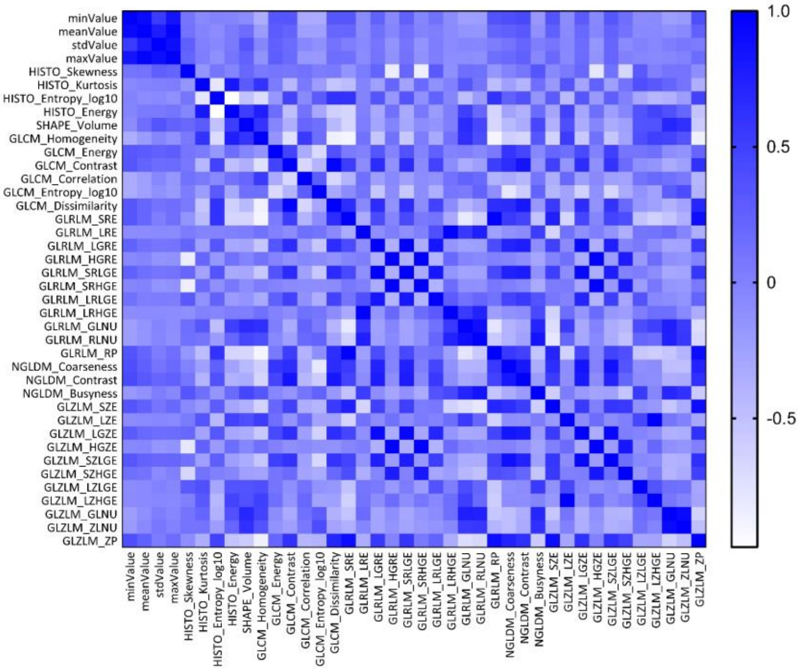

Supplementary Material 2: Heat map showing Pearson’s correlations between all pairs of features.
